# Novel and Exciting or Tried and True? Tailoring Shared Relationship Experiences to Insecurely Attached Partners

**DOI:** 10.1177/19485506251411438

**Published:** 2026-01-21

**Authors:** Kristina M. Schrage, Emily A. Impett, Mustafa Anil Topal, Cheryl Harasymchuk, Amy Muise

**Affiliations:** 1 York University, Toronto, ON, Canada; 2 University of Toronto Mississauga, ON, Canada; 3 University of Toronto, ON, Canada; 4 Carleton University, Ottawa, ON, Canada

**Keywords:** attachment avoidance, attachment anxiety, romantic relationships, self-expansion, couples

## Abstract

In the current research, we extend work on buffering attachment insecurities by examining how tailoring shared relationship experiences to attachment avoidance and anxiety is associated with relationship satisfaction. Meta-analytic results across three dyadic 21-day diary studies (*N* = 390 couples) revealed that on days when avoidantly attached individuals reported more (vs. less) novelty and excitement in their relationship, their typically lower relationship satisfaction was attenuated, in part, because they perceived greater relationship reward (i.e., intimacy and connection). Familiar and comfortable activities did not buffer avoidantly attached individuals’ relationship satisfaction. In contrast, on days when anxiously attached individuals reported more (vs. less) familiarity and comfort, their lower relationship satisfaction was fully buffered. Novel and exciting activities did not buffer anxiously attached individuals’ satisfaction. Exploratory analyses revealed that these effects also extended to partners. Thus, structuring shared time in ways that align with attachment needs may benefit the couple as a whole.

Decades of research show that attachment insecurity undermines relationship functioning. People high in attachment avoidance (who fear intimacy and closeness) and anxiety (who fear negative evaluation and abandonment) report lower satisfaction, more conflict, and greater difficulty maintaining relationships (Mikulincer & Shaver, 2016). Yet, research shows these effects can be mitigated when romantic partners tailor their behavior to meet the specific needs of insecurely attached individuals (see Overall et al., 2022, for a review). Avoidantly attached people benefit when intimacy is offered without compromising their autonomy, while anxiously attached individuals benefit from reassurance of commitment and investment. The current research extends this work by examining two forms of *shared leisure* that may help insecurely attached individuals sustain satisfaction: novel, exciting experiences (e.g., learning something new together; A. Aron et al., 2013) and familiar, comfortable experiences (e.g., cooking or watching a favorite show; Girme et al., 2014). We propose that novelty/excitement may enhance relational reward (Gere et al., 2013) for individuals high in avoidance, thereby buffering their lower satisfaction. In contrast, familiarity/comfort may reduce perceived relational threat (Gere et al., 2013) for those high in anxiety, thus improving satisfaction.

## Buffering Attachment Avoidance With Novel, Exciting Shared Experiences

Attachment avoidance is characterized by a preference for independence and discomfort with closeness, rooted in distrust of others’ support during times of need (Mikulincer & Shaver, 2019). Avoidantly attached individuals find social interactions less rewarding and emotionally fulfilling (Rognoni et al., 2008), perceiving them as dull (Tidwell et al., 1996) and less pleasant (Vrtička et al., 2012). They also report lower romantic intimacy and connection (Fraley & Davis, 1997; Spielmann et al., 2013), contributing to lower relationship satisfaction. Yet, certain autonomy-supportive rewards—such as structured self-disclosure and gentle physical touch—can mitigate this effect (Stanton et al., 2017). Similarly, feeling appreciated by a partner (Park, Impett, et al., 2019; Schrage et al., 2022), engaging in frequent sex (Little et al., 2010), and receiving expressive nonverbal cues (Schrage et al., 2020) can also dampen these effects, suggesting that although avoidant individuals tend to evade relationally rewarding experiences, such experiences can still improve their relationship satisfaction.

According to self-expansion theory, people are motivated to seek novelty/excitement to broaden their knowledge, skills, and perspectives (E. N. Aron & Aron, 1996; A. Aron et al., 2013). Self-expanding experiences enhance relationship quality beyond more familiar or mundane experiences by offering both personal and relational rewards (A. Aron et al., 2013; Harasymchuk et al., 2021) and activating the brain’s reward system (Bartels & Zeki, 2000; Fisher et al., 2010). These experiences elicit positive emotions (e.g., fun, passion, exhilaration; A. Aron et al., 2013) and foster intimacy and connection (Tsapelas et al., 2009), boosting relationship satisfaction (Harasymchuk et al., 2021). Recent evidence shows that self-change, including self-expansion, can reduce attachment avoidance (McIntyre et al., 2024). Building on this work, the current research tests whether novel, exciting experiences are especially beneficial for buffering individuals high in attachment avoidance—advancing integration between self-expansion and attachment theory in response to calls for broader theoretical synthesis in relationship science (Emery et al., 2025).

## Buffering Attachment Anxiety With Shared Familiar, Comfortable Experiences

Attachment anxiety is marked by negative self-views and chronic fears of rejection rooted in inconsistent caregiving (Ainsworth et al., 1978; Thompson, 1999). Anxiously attached individuals crave reassurance yet perceive high relational threat, often misinterpreting ambiguous cues as rejection (Alexander et al., 2001; Cassidy & Kobak, 1988; Gere et al., 2013), which heightens distress and undermines trust and satisfaction (Simpson, 1990, 2007). However, anxious individuals are highly responsive to comfort and security cues—such as affectionate touch (K. J. Kim et al., 2018), partner responsiveness (Raposo & Muise, 2021; Rholes et al., 2001), sacrifice (Murphy et al., 2022), and constructive conflict behaviors (Tran & Simpson, 2009)—that ease fears of rejection. Expressions of gratitude and exaggerated positive regard further strengthen feelings of being valued (Lemay & Dudley, 2011; Park, Impett, et al., 2019; Park, Johnson, et al., 2019), reducing insecurity and enhancing relationship satisfaction.

Most work on buffering attachment anxiety has emphasized partner reassurance, yet shared experiences may help distribute the responsibility for care while providing a pleasant experience for both partners. In fact, familiar and comfortable shared experiences can be just as beneficial for satisfaction as novel experiences (Girme et al., 2014), as they reduce relational threats—such as fears of negative evaluation—thereby fostering security (Gere et al., 2013; Mindell & Williamson, 2018). Accordingly, we predict that familiarity/comfort will buffer the link between attachment anxiety and lower satisfaction by alleviating perceived relational threat.

## The Present Research

A key contribution of this research is identifying shared experiences that address the distinct needs of avoidantly and anxiously attached individuals. Novel, exciting shared experiences may buffer avoidant individuals’ lower satisfaction by enhancing relational reward, whereas familiar, comfortable experiences may buffer anxious individuals’ lower satisfaction by reducing relational threat. Given the conceptual and statistical overlap between several of our key variables, Table 1 presents the theoretical distinctions and measures of all key constructs. We test our predictions across three dyadic daily experience studies to minimize retrospective bias and capture naturalistic experiences in couples’ daily lives (Bolger et al., 2003). Observing behaviors that occur voluntarily and with autonomy—rather than experimentally manipulated—may help identify sustainable, real-life experiences in which insecurely attached individuals and their partners are motivated to engage beyond the study. Because partners of insecurely attached individuals also tend to report lower relationship quality (e.g., Conradi et al., 2021), we explore whether these buffering effects extend to partners. Finally, we test an alternative mediation model to assess whether general positive and negative affect account for the proposed buffering effects.

**Table 1:** Descriptions of Key Constructs and Example Items

Construct	Description	Example items
Attachment avoidance	A dispositional tendency to fear closeness, value independence, and distrust others in relationships.	“I try to avoid getting too close to my partner.”
Attachment anxiety	A dispositional fear of abandonment and negative evaluation, and a desire for reassurance in relationships.	“I need a lot of reassurance that I am loved by my partner.”
Novel and exciting experiences	Shared daily activities that promote self-expansion, characterized by newness, challenge, or excitement, broaden one’s perspectives or experiences.	“How much did being with your partner result in you having new experiences?”
Familiar and comfortable experiences	Shared daily experiences that feel routine, calming, and predictable, providing comfort, stability, and a sense of security.	“Did you engage in activities that felt familiar and comfortable?” (Samples 1 and 2) “How much did your partner provide you with a source of comfort and stability today?” (Samples 1 and 2) “Today I enjoyed doing ordinary things with my partner.” (Sample 3)
Relational reward	Perceptions of intimacy, closeness, and positive connection within the relationship.	“Today, I felt closer to my partner than I’ve ever felt to somebody.”
Relational threat	Daily perceptions of judgment, rejection, or social evaluation within the relationship.	“Today, I was concerned about my partner judging me negatively.”
Relationship satisfaction	A global evaluation of contentment and fulfillment in the relationship, measured daily.	“How satisfied were you with your relationship today?”

*Note.* Familiar and comfort experiences were assessed with the same two items in Samples 1 and 2, but with a different item in Sample 3. In our validation study, we tested a factor for Familiarity and Comfort that included all three items and the items all loaded together (see p. 56 of the Online Supplemental Materials [OSM]).

## Method

We tested our hypotheses across three independent 21-day dyadic daily diary samples (*N* total = 391 couples). We explored our hypotheses in Sample 1, conducted pre-registered confirmatory analyses in Samples 2 and 3, followed by a meta-analysis across studies. Full pre-registrations, methodology, and item wordings are available on the Open Science Framework (https://osf.io/4dgfz/overview?view_only=c02c3cab1459405aa5fd8216383f1c72).

### Participants and Procedure

We recruited couples to complete a 21-day daily experience study. Recruitment was conducted via online platforms (e.g., kijiji.com, craigslist.ca, reddit.com) and paper ads in public areas (e.g., libraries and coffee shops) in Canada and the United States. Eligibility criteria included both partners being at least 18 years old, in a monogamous relationship for at least 1.5 years (Sample 1) or 2 years (Samples 2 and 3), and living together or seeing each other at least five nights per week. Interested couples were screened via phone to confirm eligibility criteria and instructed to complete the survey entries independently. Participants first completed a ∼ 50-min background questionnaire with demographic information, followed by a ∼15-min nightly questionnaire for 21 days. Compensation was $60 CAD per person in Sample 1, a $65 CAD Amazon gift card in Sample 2, and $55 CAD in Sample 3, prorated based on the number of entries completed. See Table 2 for demographic characteristics across studies. See Tables S1–S3 in the OSM for the correlations between key variables in each study.

**Table 2:** Participant Demographics by Sample

Demographic Variable	Sample 1	Sample 2	Sample 3
Gender	47.5% man, 51.2% woman, 0.8% other (not specified), 0.4% undisclosed	47.6% man, 50.7% woman, 0.3% transgender man, 0.7% nonbinary, 0.3% other (not specified), 0.3% undisclosed	44.5% man, 49.5% woman, 0.4% transgender woman, 2.9%% undisclosed
Couple Configuration	0.8% man-man, 92.6% man-woman, 1.7% woman-not specified, 0.8% man-partner undisclosed	2.0% man-man, 90.5% man-woman, 4.8% woman-woman, 0.7% woman-not specified, 0.7% man-nonbinary, 0.7% trans man-nonbinary, 0.7% woman-partner undisclosed	0.8% man-man, 89.3% man-woman, 4.1% woman-woman, 0.8% woman-trans woman, 4.1% woman-partner undisclosed, 0.8% both partners undisclosed
Age (years)	*M* = 32.63 (*SD* = 10.17, range = 20–78)	*M* = 32.60 (*SD* = 7.5, range = 20–63)	*M* = 31.53 (*SD* = 9.46, range = 19–67)
Sexual Orientation	81.4% heterosexual, 9.1% bisexual, 2.9% asexual, 2.5% lesbian, 1.7% pansexual, 0.8% gay, 0.8% queer, 0.8% not listed	82.7% heterosexual, 8.2% bisexual, 6.5% lesbian/gay, 2.6% not listed	86% heterosexual, 7% gay/lesbian, 5% bisexual, 2% not listed
Relationship Length (years)	8.50 (*SD* = 8.39, range = 1.5–58.5)	7.8 (*SD* = 5.1, range 2–35)	8.24 (*SD* = 7.10, range = 2–48)
Relationship Type	47.1% married, 29.6% cohabitating, 13.8% common law, 7.9% engaged, 0.5% other	48.3% married, 21.1% cohabitating, 29.6% common law, 1% dating	56.2% married, 21.6% engaged, 22.2% dating
Ethnic/Racial Identity	65.7% White, 4.5% Black, 8.3% East Asian, 7.4% South Asian, 1.2% Southeast Asian, 4.5% Latin American, 5.8% Bi- or multi-ethnic/racial, 1.7% Middle Eastern, 0.4% Ashkenazi Jewish, 0.4% Missing	70.7% White, 0.7% Black, 16% Asian, 1.7% Hispanic, 7.1% Multi-racial/Cultural, 3.8% identity not listed	78.3% White/European, 6.8% Latin American, 4.3% East Asian, 2.6% South Asian, 2.1% Black/African, 6.0% Bi-multi-ethnic/racial or self-identified

### Measures

#### Attachment

All samples included a baseline measure of attachment. In Samples 1 and 3, we used the 12-item Experiences in Close Relationships (ECR) Short Form (Wei et al., 2007) and, in Sample 2, the ECR-12 scale (Lafontaine et al., 2015), both rated on a 7-point scale (1 = *Strongly disagree* to 7 = *Strongly agree*). Each scale included six items assessing attachment avoidance (Sample 1: *M* = 2.03, *SD* = 0.90, α = .79; Sample 2: *M* = 2.22, *SD* = 0.99, α = .81; Sample 3: *M* = 2.04, *SD* = 0.88, α = .73) and six items assessing attachment anxiety (Sample 1: *M* = 3.40, *SD* = 1.12, α = .71; Sample 2: *M* = 3.76, *SD* = 1.53, α = .85; Sample 3: *M* = 3.45, *SD* = 1.23, α = .73).

### Daily Measures

In all samples, participants reported the extent to which they had *novel and exciting experiences* with their partner each day by rating six items adapted from the Self-Expansion Questionnaire (SEQ; Lewandowski & Aron, 2002; Muise et al., 2019). Sample 1: *M* = 3.80, *SD* = 1.88, ω = *0*.96; Sample 2: *M* = 3.67, *SD* = 1.50 ω = 0.96; Sample 3: *M* = 3.07, *SD* = 1.79; ω = 0.96) on a 7-point scale (1 = *Not very much* to 7 = *Very much*). In Samples 1 and 2, *familiar and comfortable* ***experiences*** were assessed with two items (Sample 1: *M* = 5.80, *SD* = 1.41, *r* = 0.71; Sample 2: *M* = 5.68, *SD* = 1.17, *r* = 0.62), rated on a 7-point scale (1 = *Not very much* to 7 = *Very much*). In Sample 3, we used a single item adapted for the daily context from Reis et al. (2014) (*M* = 5.96, *SD* = 1.37; 1 = *Strongly disagree to* 7 = *Strongly agree*). In a separate study, we asked participants for examples of both novel and exciting and familiar and comfortable experiences, and we coded them using thematic analysis (for the themes and examples see p. 59 in the OSM).

We assessed *relational reward and threat* in Samples 1 and 3 using an abbreviated version of the Social Threat and Reward Scale (Spielmann et al., 2012), adapted for daily use. Three items measured daily perceptions of relational reward (Sample 1: *M* = 4.42, *SD* = 0.85, ω = 0.90; Sample 3: *M* = 4.45, *SD* = 0.78, ω = 0.83) and three items assessed relational threat (Sample 1: *M* = 1.53, *SD* = 0.95, ω = 0.90; Sample 3: *M* = 1.46, *SD* = 0.79, ω = 0.80). All items were rated on a 5-point scale (1 = *Strongly disagree* to 5 = *Strongly agree*). Relational reward and threat were not assessed in Sample 2. In all samples, we measured ***relationship satisfaction*** with a single face-valid item adapted for daily use from the Perceived Relationship Quality Components scale (Fletcher et al., 2000) (Sample 1: *M* = 6.04, *SD* = 1.25; Sample 2: *M* = 5.99, *SD* = 1.17; Sample 3: *M* = 6.13, *SD* = 1.17), rated on a 7-point scale (1 = *Not at all* to 7 = *Extremely*). Previous research demonstrated that reward and threat are distinct from relationship satisfaction (Gere et al., 2013). However, given that we assessed several positively valenced relational constructs in the current study, we conducted a separate validation study to assess the distinctiveness between the different types of experiences, relational reward, and relationship satisfaction. Using confirmatory factor analysis, two-factor models fit significantly better than one-factor models in all cases, Δχ^2^ tests *p* < .001; see pp. 56–58 of the OSM for details.

### Data Analytic Approach

We first conducted analyses within each sample (see Tables S11, S14, and S17 in the OSM; see also Supplemental Figures S1–S12 for interactive effects across studies). Using SPSS 28, we ran two-level cross-classified multilevel models with random intercepts. Participants were nested within couples, and days and couples were crossed to account for both partners completing surveys on the same day (Kenny et al., 2006). To test our primary buffering hypotheses, we examined cross-level interactions between grand mean-centered attachment variables and within-person centered daily shared experience variables. For control purposes, we included grand mean-centered aggregates of the daily experience variables and their interactions with grand-mean-centered attachment variables.

Next, to test whether perceptions of relational reward and threat explain how different types of shared experiences buffer insecurely attached people from experiencing lower satisfaction, we analyzed data from Samples 1 and 3 (which included reward/threat measures). We tested the main effects and interactive effects of attachment (avoidance and anxiety) and shared experiences (novel/exciting and comfortable/familiar) at both within- and between-person levels on reward and threat. We then added reward and threat and their interactions with attachment (avoidance and anxiety) at both within- and between-person levels to predict relationship satisfaction.

After obtaining sample-specific results, we meta-analyzed findings in R (v4.2.1) using the metafor package (Viechtbauer & Viechtbauer, 2015). We used a random-effects approach (e.g., Schmidt et al., 2009) to compute semi-partial correlation coefficients (Aloe, 2014) and 95% confidence intervals for all main effects and interactions. For mediation, we meta-analyzed each pathway and estimated indirect effects using the Monte Carlo Method to Assess Mediation with 20,000 resamples to compute 95% confidence intervals (Selig & Preacher, 2008). We calculated *R*^2^ estimates following Nakagawa and Schielzeth’s (2012) approach and using the partR2 package (Stoffel et al., 2021). We input t-statistics from the full models (Studies 1–3; see Tables S11, S14, and S17 in the OSM). Our key predictions focus on cross-level interactions between attachment and within-person fluctuations in daily shared experiences (see Table 3); between-person effects are shown in Table S4 of the OSM. Sample 2 was collected during a time when many of the participants were abiding by stay-at-home orders during the COVID-19 pandemic, and the findings in Sample 2 diverged from predictions (see OSM Table S14). Therefore, we also conducted leave-one-out analyses to determine if any specific study was driving or detracting from an effect.

**Table 3:** Attachment Insecurity and Shared Experiences Predicting Daily Relationship Satisfaction

	Relationship satisfaction				
	Estimate	*SE*	*z*	*p*	95% CI
Avoidance (Between-Person)	−0.13	0.03	−5.02	<.001	[−0.18, −0.08]
Anxiety (Between-Person)	−0.09	0.03	−2.72	.007	[−0.15, −0.02]
Novel and Exciting (Within-Person)	0.13	0.02	5.70	<.001	[0.08, 0.17]
Familiar and Comfortable (Within-Person)	0.30	0.02	15.02	<.001	[0.26, 0.34]
Avoidance (Between-Person) × Novel and Exciting (Within-Person)	0.02	0.01	3.99	<.001	[0.01, 0.04]
Avoidance (Between-Person) × Familiar and Comfortable (Within-Person)	0.01	0.02	0.38	.702	[−0.03, 0.05]
Anxiety (Between-Person) × Novel and Exciting (Within-Person)	0.02	0.01	1.91	.055	[−0.0004, 0.04]
Anxiety (Between-Person) × Familiar and Comfortable (Within-Person)	0.02	0.01	3.99	<.001	[0.01, 0.04]

*Note.* Effects are the meta-analytic effects across all three samples derived from a multilevel model in which all predictors were simultaneously entered to predict the outcome. Estimate coefficients are derived from unstandardized effects.

## Results

Consistent with past work, people higher in attachment avoidance and anxiety reported lower relationship satisfaction (see Table 3). In addition, on days when people reported more novelty/excitement and comfort/familiarity than they typically did across the 21-day period, they reported higher relationship satisfaction, suggesting that both types of activities are linked to greater daily satisfaction. However, our main predictions focused on interactions between attachment orientation (anxiety and avoidance) and the type of shared couple activity.

### Buffering Attachment Avoidance

**Figure 1:**
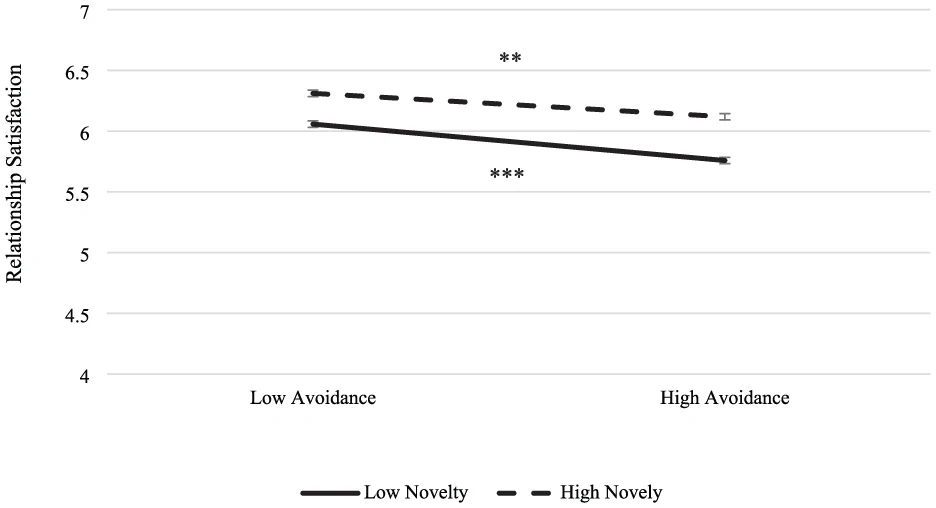
The Moderating Effect of Novelty and Excitement on the Link Between Attachment Avoidance and Daily Relationship Satisfaction **p<.01 ***p<.001.

Consistent with our prediction, meta-analytic results revealed a significant interaction between attachment avoidance and novelty/excitement predicting daily relationship satisfaction (see Figure 1). To confirm the pattern of effects, we meta-analyzed the simple effects of attachment avoidance at low (−1 *SD*) and high (+1 *SD*) levels of daily novel/exciting shared experiences. When people reported less novelty/excitement than usual, avoidantly attached individuals reported lower relationship satisfaction than those lower in avoidance (*sr*^1^ = −.16, *SE* = .03, *p* < .001, 95% CI = [−0.21, −0.11]). However, when people reported more novelty/excitement than usual, the negative link between avoidance and satisfaction was attenutated, though still significant (*sr* = −.09, *SE* = .03, *p =* .001, 95% CI = [−0.14, −0.04]). We also decomposed the interaction at low (−1 *SD*) and high (+1 *SD*) levels of attachment avoidance and found that for people low in attachment avoidance, there was a significant association between novelty/excitement and relationship satisfaction (*sr* = .07, *SE* = .02, *p*<.001, 95% *CI*[.04, 0.11]), but the link was stronger for those high in attachment avoidance (*sr* = .10, *SE* = 0.02, *p* < .001, 95% CI = [0.08, 0.13]). This interaction remained significant after controlling for previous day satisfaction (*sr* = .02, *SE* = 0.01, *p* = .003, 95% CI = [−0.14, −0.04]; see Table S8 in OSM). Leave-one-out analyses showed that results did not change when any sample was excluded. Overall, higher daily novelty and excitement partially buffered avoidantly attached individuals’ typically lower relationship satisfaction.

#### Discriminant Analyses

There was no significant interaction between attachment avoidance and daily familiarity/comfort in predicting relationship satisfaction (see Table 3). A leave-one-out analysis showed the pattern of results remained unchanged when any sample was excluded.

#### Mediation Analyses

**Figure 2:**
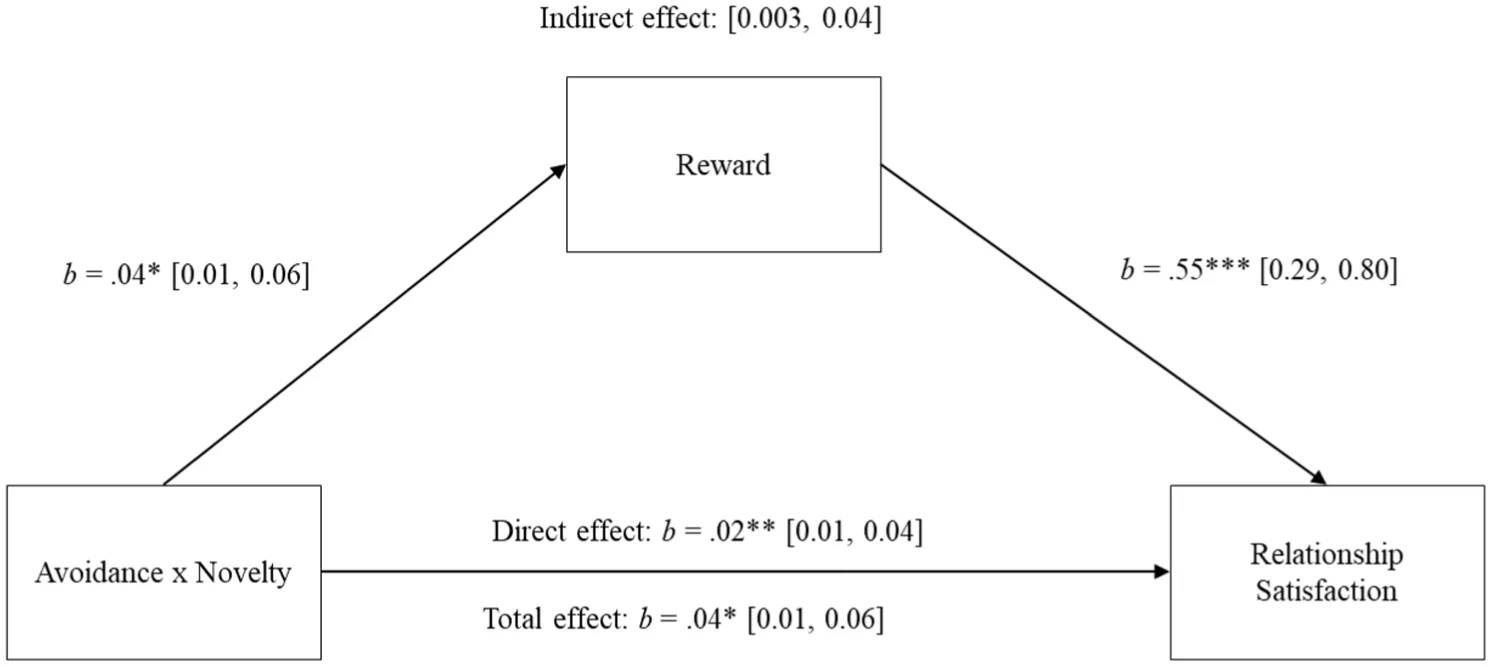
The Indirect Effect of the Interaction Between Attachment Avoidance and Novelty and Excitement Predicting Relationship Satisfaction Through Relational Reward Unstandardized b Values Obtained From Meta-Analyzed Results From Studies 1 and 3)

We expected shared novel experiences with a partner would buffer avoidantly attached individuals from experiencing lower daily relationship satisfaction by enhancing their typically low levels of relational reward. Indeed, there was a significant interaction between attachment avoidance and daily novelty/excitement predicting relationship reward (*sr* = .05, *SE* = .02, *p* = .013, 95% CI = [0.01, 0.09]; see Tables S5 and S6 in the OSM for full partial correlation coefficients for each path). On days when people reported lower (−1 *SD*) novelty/excitement than usual, avoidantly attached individuals reported lower relationship reward (*sr* = −.06, *SE* = .01, *p*<.001, 95% CI = [−0.08, −0.04]). On days with higher novelty/excitement, the negative link between avoidance and relational reward was reduced (*sr* = −.03, *SE* = .01, *p*<.001, 95% CI = [−0.04, −0.01]). Decomposing the other simple slopes, for people low in attachment avoidance, novelty/excitement was associated with greater relational reward (*sr* = .05, *SE* = .01, *p*<.001, 95% CI = [0.04, 0.07], but the link was stronger for those higher in attachment avoidance (*sr* = .12, *SE* = .03, *p*<001, 95% CI = [0.05, 0.18]). There was no significant interaction between attachment avoidance and novelty/excitement predicting relational threat (*sr =* .03, *SE =* .03, *p* = .1681, 95% CI = [−0.0164, 0.0941]). To compute the indirect effect of the interaction between attachment avoidance and novelty/excitement predicting relationship satisfaction via relational reward, we converted the results of the estimates into unstandardized *b* values and used these to obtain the 95% confidence intervals [.003, .04], which indicated a significant pathway (see Figure 2).

### Buffering Attachment Anxiety

**Figure 3:**
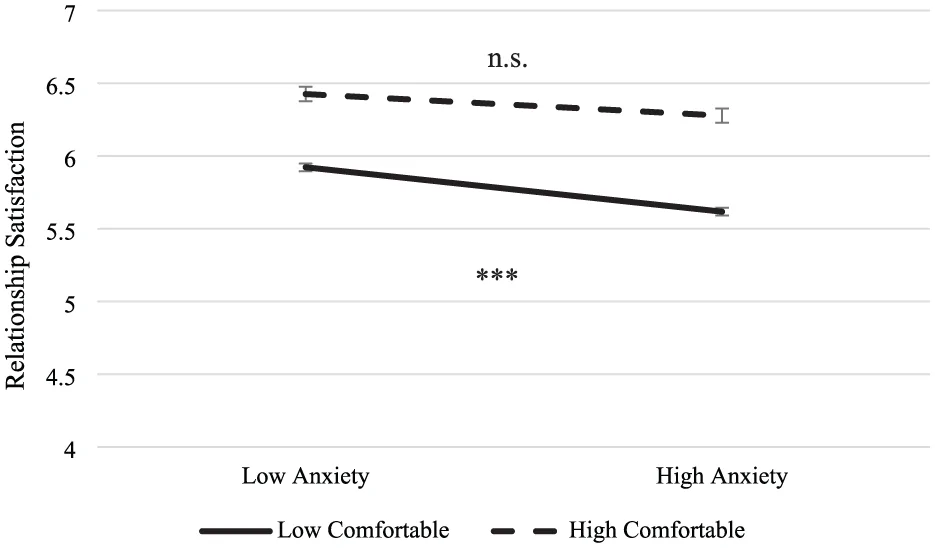
The Moderating Effect of Familiar and Comfortable Experiences on the Link Between Attachment Anxiety and Daily Relationship Satisfaction ***p<.001.

Consistent with our prediction, meta-analytic results revealed a significant interaction between attachment anxiety and familiarity/comfort predicting daily relationship satisfaction (see Figure 3). Simple effects analyses showed that on days with fewer familiar/comfortable experiences than usual, anxiously attached individuals reported lower satisfaction (*sr* = −.13, *SE* = .03, *p* < .001, 95% CI = [−0.18, −0.08]). However, on days with more familiar/comfortable experiences, the negative link between anxiety and satisfaction was no longer significant (*sr* = −.03, *SE* = .05, *p* = .514, 95% CI = [−0.14, 0.07]). Decomposed the other way, for people lower in attachment anxiety, more familiar/comfortable experiences were associated with higher relationship satisfaction (*sr* = .08, *SE* = .01, *p*<.001, 95% CI = [0.06, 0.10]), but there was even stronger association for those high in attachment anxiety (*sr* = .19, *SE* = .03, *p*<.001, 95% CI = [0.13, 0.25]). The interaction remained significant when controlling for prior-day satisfaction (*sr* = .04, *SE* = 0.02, *p* = .027, 95% CI = [0.004, 0.07]). Leave-one-out analyses showed the effect was strongest when excluding Study 2 (*sr* = .05, *SE* = .01, *p*<.001, 95% CI = [0.03, 0.06]), and became nonsignificant when excluding Study 1 (*sr* = .03, *SE* = .02, *p* = .245, 95% CI = [−0.02, 0.08]) or Study 3 (*sr* = .02, *SE* = .02, *p* = .217, 95% CI = [−0.01, 0.05]). These results suggest that Studies 1 and 3 contributed to the buffering effect, while Study 2 weakened the effect. Overall, these results indicate that familiar/comfortable experiences fully buffered anxiously attached individuals’ lower relationship satisfaction.

#### Discriminant Analyses

Meta-analytic results showed a nonsignificant interaction between attachment anxiety and daily novelty/excitement predicting relationship satisfaction (see Table 3). Leave-one-out analyses indicated that the interaction became significant when Study 3 was removed (*sr* = .03, *SE* = .01, *p* = .030, 95% CI = [0.003, 0.05]), but remained nonsignificant when excluding Study 1 (*sr* = .02, *SE* = .02, *p* = .191, 95% CI = [−0.01, 0.06]) or Study 2 (*sr* = .01, *SE* = .01, *p* = .189, 95% CI = [−0.005, 0.02]). Overall, these results suggest that anxiously attached individuals’ relationship satisfaction is more reliably buffered by familiarity/comfort than novelty/excitement.

#### Mediation Analyses

We expected that familiar, comfortable experiences would buffer anxiously attached individuals from lower daily relationship satisfaction by reducing their typically high levels of relational threat. Contrary to predictions, the interaction between attachment anxiety and daily familiarity/comfort did not significantly predict relational threat (*sr* = .01, *SE* = .01, *p* = .131, 95% CI = [0.004, 0.03]), nor did it predict relational reward (*sr* = .01, *SE* = .01, *p* = .244, 95% CI = [0.01, 0.03]). Therefore, we did not find evidence that relational threat or reward explained the buffering effect for those higher in anxious attachment.

### Buffering a Partner’s Attachment Insecurity

In a set of non-preregistered exploratory analyses, we tested whether these benefits extended to the romantic partner. That is, whether daily novel/exciting experiences buffered people’s lower relationship satisfaction associated with their partner’s attachment avoidance, and whether daily familiar/comfortable experiences buffered lower relationship satisfaction associated with their partner’s attachment anxiety. We used the same basic model as the primary analyses, adding partner attachment avoidance and anxiety as predictors, along with cross- and same-level interactions between partner attachment (avoidance, anxiety) and actor-reported daily shared experiences (novel/exciting, comfortable/familiar). We tested cross-level interactions between between-person partner attachment and within-person daily experiences in predicting actor daily relationship satisfaction. As with our main analyses, models were tested separately in each sample and meta-analyzed across studies. Our primary focus was on cross-level interactions between partner attachment and shared experiences, shown in Table 4. Full model results, including the interactions with partner attachment and between-person shared experiences, are in Table S7 of the OSM.

**Table 4:** Actor and Partner Attachment Insecurity and Actor Shared Experiences Predicting Actor Daily Relationship Satisfaction

	Relationship satisfaction				
	Estimate	*SE*	*z*	*p*	95% CI
Actor Avoidance (Between-Person)	−.12	.03	−4.59	<.001	[−0.17, −0.07]
Partner Avoidance (Between-Person)	−.06	.05	−1.17	0.243	[−0.16, 0.04]
Actor Anxiety (Between-Person)	−.09	.05	−1.89	.059	[−0.17, 0.003]
Partner Anxiety (Between-Person)	−.09	.05	−1.81	.071	[−0.18, 0.01]
Actor Novel and Exciting (Within-Person)	0.13	.02	6.11	<.001	[0.09, 0.17]
Actor Familiar and Comfortable (Within-Person)	0.30	.02	15.54	<.001	[0.26, 0.34]
Actor Avoidance (Between-Person) × Novel and Exciting (Within-Person)	.04	.01	3.47	<.001	[0.01, 0.04]
Partner Avoidance (Between-Person) × Novel and Exciting (Within-Person)	.02	.01	2.34	.019	[0.003, 0.03]
Actor Avoidance (Between-Person) × Familiar and Comfortable (Within-Person)	−.003	.02	−0.14	0.890	[−0.05, 0.04]
Partner Avoidance (Between-Person) × Familiar and Comfortable (Within-Person)	−.01	.02	−0.53	0.596	[−0.04, 0.02]
Actor Anxiety (Between-Person) × Novel and Exciting (Within-Person)	.01	.01	1.23	0.220	[−0.01, 0.04]
Partner Anxiety (Between-Person) × Novel and Exciting (Within-Person)	−.005	.01	−0.65	0.514	[−0.02, 0.01]
Actor Anxiety (Between-Person) × Familiar and Comfortable (Within-Person)	.04	.02	2.11	.035	[0.01, 0.07]
Partner Anxiety (Between-Person) × Familiar and Comfortable (Within-Person)	.04	.01	6.59	<.001	[0.03, 0.05]

*Note.* Estimates are the meta-analytic effects across all three samples derived from multi-level models in which all predictors were simultaneously entered to predict the outcome. Estimate coefficients are derived from unstandardized effects.

**Figure 4:**
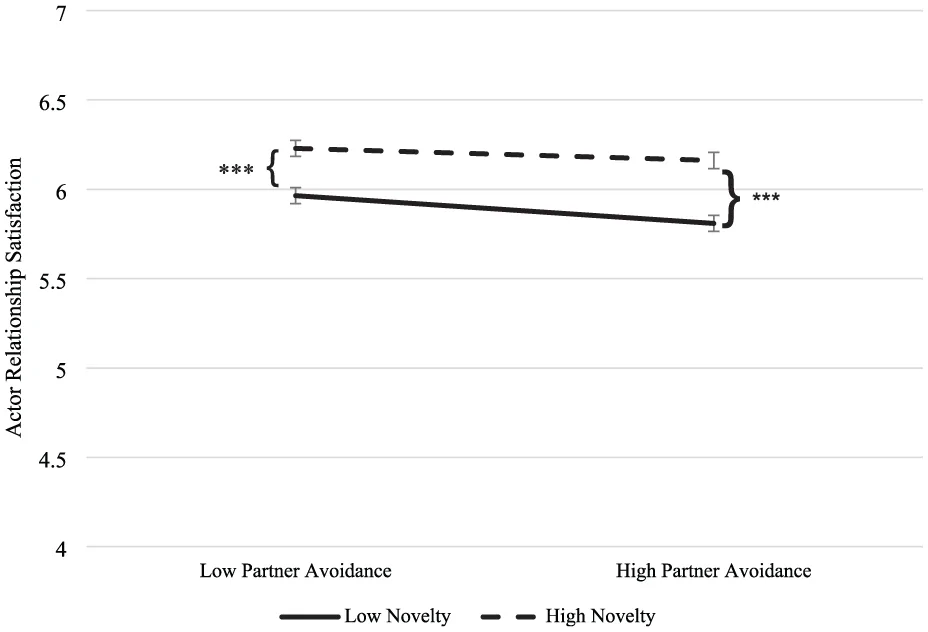
The Moderating Effect of Actor Novelty and Excitement on the Link Between Partner Attachment Avoidance and Actor Daily Relationship Satisfaction

Results indicated a significant interaction between the partner’s attachment avoidance and actor-reported novel/exciting experiences predicting the actor’s daily relationship satisfaction (see Figure 4), but no significant interaction with familiar and comfortable experiences (see Table 4). Simple slopes analyses at 1 *SD* below and above the mean of actor-reported novelty/excitement showed no significant effect of partner avoidance on actor daily satisfaction at lower novelty/excitement (*sr* = −.08, *SE* = .06, *p* = .150), suggesting that there was no negative effect to buffer. Similarly, no effect emerged at higher levels of novelty/excitement (*sr* = −.04, *SE* = .04, *p* = .330). Decomposing the interaction the other way showed that actor-reported novelty/excitement predicted greater satisfaction for partners lower in attachment avoidance (*sr* = .07, *SE* = .01, *p*<.001), and even more so for partners higher in avoidance (*sr* = .10, *SE* = .02, *p* <.001). Thus, the benefits of novel and exciting experiences for relationship satisfaction were especially pronounced when the partner was more avoidantly attached.

**Figure 5:**
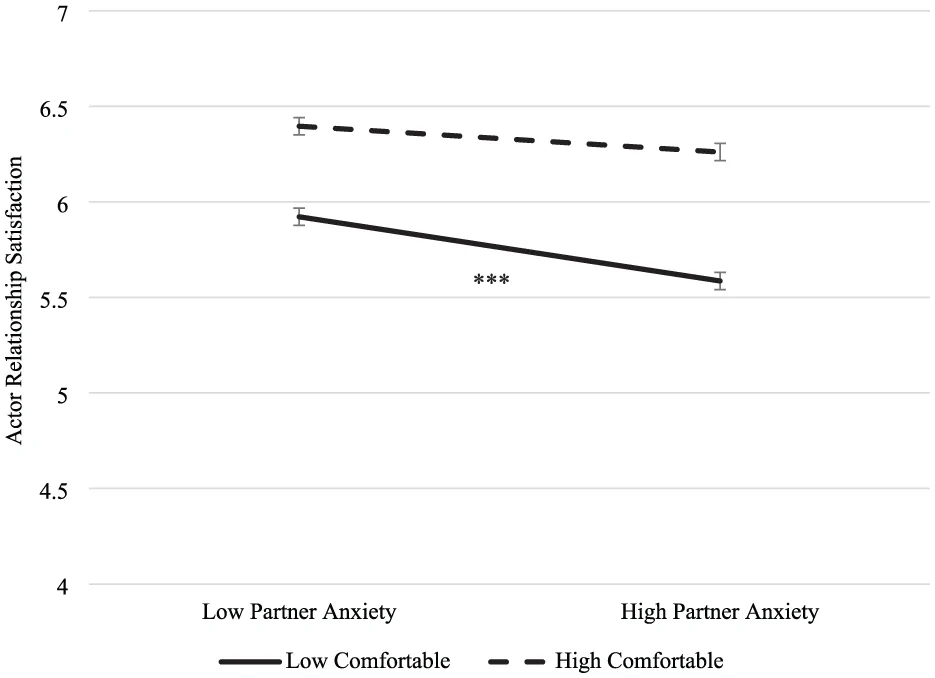
The Moderating Effect of Actor Familiar and Comfortable Experiences on the Link Between Partner Attachment Anxiety and Actor Daily Relationship Satisfaction ***p<.001.

Results indicated a significant interaction between the partner’s attachment anxiety and actor-reported familiar/comfortable experiences predicting the actor’s daily relationship satisfaction (see Figure 5), but no significant interaction with novel/exciting experiences (see Table 4). Simple slopes analyses indicated that at lower levels of familiarity/comfort, partner attachment anxiety was linked to lower actor satisfaction (*sr* = −.14, *SE* = .04, *p*<.001). At higher levels, this negative effect was attenuated and nonsignificant (*sr* = −.05, *SE* = .07, *p* = .493). Decomposing the simple slopes in the other direction, when the partner was lower in attachment anxiety, familiarity/comfort was associated with greater actor relationship satisfaction (*sr* = .19, *SE* = .02, *p*<.001, 95% CI = [0.15, 0.24]). However, when the partner was higher in attachment anxiety, the link between familiarity/comfort and the actor’s relationship satisfaction was even stronger (*sr* = .24, *SE* = .02, *p*<.001, 95% CI = [0.21, 0.27]. These findings suggest that familiar and comfortable experiences fully buffer people from the negative association of a partner’s attachment anxiety on relationship satisfaction.

### Testing an Alternative Model

We also tested whether the buffering effects of shared experiences on attachment insecurity could be accounted for by more general positive and negative emotions experienced each day (See Table S9 and S10 in the OSM; data only available and tested in Sample 3). The interaction between attachment avoidance and novelty/excitement did not predict daily positive emotions (*b* = .01, *SE* = .02, *t*(4218) = 0.69, *p* = .490), suggesting this effect is not due to broader positive affect. However, there was a significant interaction between attachment anxiety and daily familiarity/comfort predicting daily negative emotion (*b* = −.04, *SE* = .01, *t*(4434) = −3.31, *p*<.001). Simple slopes analyses showed that at low levels of comfort/familiarity, attachment anxiety was associated with greater negative affect (*b* = 0.30, *SE* = .05, *t*(265) = 6.60, *p*<.001), but at high levels, this association was weaker (*b* = 0.25, *SE* = .05, *t*(265) = 5.41, *p*<.001. The indirect effect predicting daily relationship satisfaction was significant (95% CI = [0.001, 0.007]), suggesting that the buffering effect of familiarity/comfort on relationship satisfaction for anxiously attached individuals can be partly explained by them experiencing less negative affect overall when engaging in these experiences with their partner.

## General Discussion

The present research advances our understanding of how shared experiences can buffer attachment insecurities when they align with individuals’ attachment-related needs. Across a meta-analysis of three dyadic daily experience studies, avoidantly attached individuals’ lower relationship satisfaction was partially buffered on days with more (vs. less) novel, exciting experiences, but did not differ based on their engagement in familiar, comfortable experiences. In contrast, anxiously attached individuals’ lower relationship satisfaction was fully buffered on days with more (vs. less) familiar, comfortable experiences. Novel, exciting experiences also buffered anxious individuals’ low relationship satisfaction, but this effect was weaker and less consistent than the buffering effect of familiar, comfortable experiences.

We found that novel, exciting experiences buffered avoidantly attached individuals’ typically low relationship satisfaction by enhancing their perceptions of relational reward (Gere et al., 2013), which in turn was linked to greater daily relationship satisfaction. Although avoidantly attached individuals often downplay their needs for intimacy and connection (Mikulincer & Shaver, 2016), shared novel, exciting experiences may evoke positive emotions that temporarily override negative working models of others (Stanton et al., 2017). Such experiences may provide “masked” intimacy—connection embedded within enjoyable, goal-directed activity—that feels more acceptable to those who typically avoid overt displays of intimacy (Stanton et al., 2017).

Although familiar, comfortable experiences buffered anxiously attached individuals’ lower relationship satisfaction, this effect did not operate through reduced relational threat as expected. Despite elevated threat perceptions among anxiously attached individuals (Gere et al., 2013), follow-up analyses showed that familiar, comfortable experiences instead partially buffered against broader negative affect, which in turn predicted greater relationship satisfaction. An alternative explanation is that such experiences may enhance security, which was not assessed in the current studies, rather than reduce threat, consistent with evidence that security-enhancing interactions can both buffer dissatisfaction and promote greater attachment security over time (Kumashiro & Arriaga, 2020).

Exploratory analyses showed that familiar experiences buffered the lower satisfaction associated with having a partner high in attachment anxiety, and that the benefits of novel experiences for relationship satisfaction were especially pronounced when a partner was higher (vs. lower) in attachment avoidance. Although exploratory, these findings are theoretically meaningful and suggest that a partner’s attachment orientation shapes the conditions under which shared experiences are most beneficial. That is, the benefits of tailoring shared activities extend beyond the insecure individual to their partner’s satisfaction, highlighting the inherently dyadic nature of attachment buffering. These results suggest that interventions helping couples structure shared time around each partner’s attachment needs may enhance relationship satisfaction for both partners.

### Theoretical Contributions

This research extends previous work by showing how different types of shared experiences can buffer attachment insecurities and mitigate negative relationship outcomes. Avoidantly attached individuals, who value autonomy and independence (Overall et al., 2022), may benefit most from novel, exciting experiences in which intimacy is embedded within fun, low-pressure contexts. In contrast, anxiously attached individuals may gain a greater sense of agency and security from familiar, comfortable experiences. According to the Attachment Security Enhancement Model (ASEM; Kumashiro & Arriaga, 2020), fostering agency can reduce anxious individuals’ dependence and fears of rejection, thereby enhancing relationship satisfaction. Future research should test whether familiar experiences bolster security for anxiously attached individuals, explaining associations with satisfaction.

These findings also extend self-expansion theory by identifying attachment avoidance as a moderator of the benefits of shared novel, exciting experiences. While prior research has linked self-expanding experiences to improved relationship quality (A. Aron et al., 2022), few studies have identified the conditions that enhance or diminish these benefits. This study provides the first evidence that self-expanding (i.e., novel, exciting) experiences may be especially beneficial for avoidantly attached individuals. By demonstrating when and for whom self-expansion is most beneficial, these findings refine our understanding of how shared novelty interacts with individual differences in attachment, offering new insights into strategies for sustaining relationship satisfaction.

### Limitations and Future Directions

Although our daily diary data helped reduce retrospective bias (Bolger et al., 2003), our correlational data prevent causal conclusions. Relationship satisfaction could boost novel (or familiar) activities rather than the reverse, or the link could be reciprocal. Experimental designs could clarify the causal direction, but since these experiences arose naturally, it is unclear whether instructing insecurely attached individuals to engage in specific activities would yield the same benefits. Avoidantly attached individuals, who value autonomy, may resist engaging in prescribed experiences (Mikulincer & Shaver, 2016), while anxiously attached individuals may need reassurance that their partner’s participation is genuine rather than obligatory. Future experiments could explore these possibilities.

There were some inconsistencies in the findings across the individual samples, which highlight directions for future work. The pattern of findings in Sample 2 diverged from our predictions. Although this may in part reflect the unique circumstances of the COVID-19 pandemic (which constrained couples’ leisure activities), it also raises the possibility that contextual constraints may shift which types of experiences are beneficial. For example, in a period when opportunities for novelty were severely limited, familiar and comfortable experiences may have taken on heightened importance for sustaining satisfaction. The contextual factors that shift the benefits of different types of leisure activities are a direction for future research.

It is important to acknowledge that while both novelty/excitement and familiarity/comfort were associated with higher relationship satisfaction, the strength of the effect was nearly three times greater for familiarity/comfort (*sr* = .30) than novelty/excitement (*sr* = .13). Therefore, although more (vs. less) novel experiences were beneficial for people high in attachment avoidance, familiar experiences were associated with greater satisfaction as well (the strength of the associated just did not differ based on attachment avoidance). Also, although we conducted additional analyses to demonstrate the distinctiveness of the key constructs, familiar experiences, reward, and relationship satisfaction were highly correlated and could be tapping a general relationship construct (J. J. Kim et al., 2025). Future work that goes beyond self-report methods, including observational coding of couples’ shared activities or interactions, could address this limitation.

We also do not know how the daily experiences were initiated, which may influence their effectiveness in buffering lower relationship quality. Avoidantly attached individuals may benefit more when they initiate novel experiences, while anxiously attached individuals may feel more secure when their partner initiates familiar ones. It is also important to note that people characterized as high in attachment avoidance in the current samples nevertheless scored below the midpoint of the scale, so it is not clear whether the results generalize to people at truly high levels. In addition, although we observed daily benefits, the long-term impact of shared experiences remains unknown. Since positive relationship experiences can foster attachment security (Bayraktaroglu et al., 2023; Kumashiro & Arriaga, 2020), future longitudinal research should examine whether repeated shared experiences sustain relationship satisfaction and reduce insecurity.

## Conclusion

Insecurely attached individuals often struggle to maintain satisfying relationships. This research shows that engaging in shared relationship experiences that meet their unique, attachment-related needs can help. For people who are more avoidant and tend to shy away from closeness, engaging in novel, exciting activities with their partner boosts relationship satisfaction partly because it makes the relationship feel more rewarding. For people who are more anxious and often worry about negative evaluation and abandonment, spending time together doing something familiar and comfortable helps them feel more satisfied. These findings show that sharing the right kind of experience with a partner can strengthen relationships, even for people who struggle with intimacy and connection.

## Supplemental Material

sj-docx-1-spp-10.1177_19485506251411438 – Supplemental material for Novel and Exciting or Tried and True? Tailoring Shared Relationship Experiences to Insecurely Attached Partners Supplemental material, sj-docx-1-spp-10.1177_19485506251411438 for Novel and Exciting or Tried and True? Tailoring Shared Relationship Experiences to Insecurely Attached Partners by Kristina M. Schrage, Emily A. Impett, Mustafa Anil Topal, Cheryl Harasymchuk and Amy Muise in Social Psychological and Personality Science

## Notes

1. *sr* represents a semi-partial correlation, which is standard in meta-analysis.
