# Supplementary material for: Novel and Exciting or Tried and True? Tailoring Shared Relationship Experiences to Insecurely Attached Partners
Source: Soc Psychol Personal Sci. 2026 Jan 21;17(8):980–92. doi: 10.1177/19485506251411438 (PMC13588490; doi:10.1177/19485506251411438)
Supplement: Supplementary file 1 [file sj-docx-1-spp-10.1177_19485506251411438.docx]

Online Supplemental Materials for “Novel and Exciting or Tried and True? Tailoring Shared Relationship Experiences to Insecurely Attached Partners”

Table of Contents

[Table S1	3](#_Toc206696036)

[Bivariate correlations among all variables in Sample 1	3](#_Toc206696037)

[Table S2	5](#_Toc206696038)

[Bivariate correlations among all variables in Sample 2	5](#_Toc206696039)

[Table S3	6](#_Toc206696040)

[Bivariate correlations among all variables in Sample 3	6](#_Toc206696041)

[Table S4	7](#_Toc206696042)

[Meta-Analyzed Effects of Attachment Insecurity and Shared Experiences Predicting Daily Relationship Satisfaction	7](#_Toc206696043)

[Table S8	16](#_Toc206696044)

[Meta-Analyzed Effects of Attachment Insecurity and Shared Experiences Predicting Daily Relationship Satisfaction Controlling for Previous Day Outcome (Yesterday’s Relationship Satisfaction)	16](#_Toc206696045)

[Figure S1.	39](#_Toc206696046)

[The moderating effect of novelty and excitement on the link between attachment avoidance and daily relationship satisfaction in Studies 1 (A.), 2 (B.), and 3 (C.).	39](#_Toc206696047)

[Figure S2.	41](#_Toc206696048)

[The moderating effect of familiarity and comfort on the link between anxiety and daily relationship satisfaction in Studies 1 (A.), 2 (B.) and 3 (C.).	41](#_Toc206696049)

[Figure S3.	43](#_Toc206696050)

[The moderating effect of familiarity and comfort on the link between attachment avoidance and daily relationship satisfaction in Studies 1 (A.), 2 (B.), and 3 (C.).	43](#_Toc206696051)

[Figure S4.	45](#_Toc206696052)

[The moderating effect of novelty and excitement on the link between attachment anxiety and daily relationship satisfaction in Studies 1 (A.), 2 (B.), and 3 (C.).	45](#_Toc206696053)

[Figure S5.	47](#_Toc206696054)

[The moderating effect of novelty and excitement on the link between attachment avoidance and daily relational reward in Studies 1 (A.) and 3 (B.).	47](#_Toc206696055)

[Figure S6.	49](#_Toc206696056)

[The moderating effect of novelty and excitement on the link between attachment avoidance and daily relational threat in Studies 1 (A.) and 3 (B.).	49](#_Toc206696057)

[Figure S7.	50](#_Toc206696058)

[The moderating effect of familiarity and comfort on the link between attachment avoidance and daily relational reward in Studies 1 (A.) and 3 (B.).	50](#_Toc206696059)

[Figure S8.	51](#_Toc206696060)

[The moderating effect of familiarity and comfort on the link between attachment avoidance and daily relational threat in Studies 1 (A.) and 3 (B.).	51](#_Toc206696061)

[Figure S9.	52](#_Toc206696062)

[The moderating effect of familiarity and comfort on the link between attachment anxiety and daily relational threat in Studies 1 (A.) and 3 (B.).	52](#_Toc206696063)

[Figure S10.	53](#_Toc206696064)

[The moderating effect of familiarity and comfort on the link between attachment anxiety and daily relational reward in Studies 1 (A.) and 3 (B.).	53](#_Toc206696065)

[Figure S11.	54](#_Toc206696066)

[The moderating effect of novelty and excitement on the link between attachment anxiety and daily relational reward in Studies 1 (A.) and 3 (B.).	54](#_Toc206696067)

[Figure S12.	55](#_Toc206696068)

[The moderating effect of novelty and excitement on the link between attachment anxiety and daily relational threat in Studies 1 (A.) and 3 (B.).	55](#_Toc206696069)

[Validation Study	56](#_Toc206696070)

[Testing the Distinctiveness of our Key Constructs	56](#_Toc206696071)

[Coding Examples of Familiar-Comfortable and Novel-Exciting Experiences	59](#_Toc206696072)

[References	60](#_Toc206696073)

| Table S1 |  |  |  |  |
| --- | --- | --- | --- | --- |
| Bivariate correlations among all variables in Sample 1 | | | | |

|  | 1 | 2 | 3 | 4 | 5 | 6 |
| --- | --- | --- | --- | --- | --- | --- |
| 1. Attachment Avoidance | — |  |  |  |  |  |
| 2. Attachment Anxiety | .424*** | — |  |  |  |  |
| 3. Novelty and Excitement | .036 | .059 | — |  |  |  |
| 4. Familiarity and Comfort | -.454*** | -.338*** | .273*** | — |  |  |
| 5. Relational Reward | -.490*** | -.335*** | .383*** | .758*** | — |  |
| 6. Relational Threat | .370*** | .313*** | .150* | -.386*** | -.314*** | — |
| 7. Relationship Satisfaction | -.418*** | -.312*** | .301*** | .844*** | .842*** | -.342*** |

*Note.* Attachment avoidance and anxiety were assessed at background. All other variables were assessed at the daily level. Correlations with daily variables are with the between-person aggregates across the entire 21-day period of the study.

*** *p* < .001

# **Table S2**

## **Bivariate correlations among all variables in Sample 2**

|  | 1 | 2 | 3 | 4 |
| --- | --- | --- | --- | --- |
| 1. Attachment Avoidance | — |  |  |  |
| 2. Attachment Anxiety | .106† | — |  |  |
| 3. Novelty and Excitement | -.066 | .037 | — |  |
| 4. Familiarity and Comfort | -.300*** | .040 | .248*** | — |
| 5. Relationship Satisfaction | -.372*** | -.108† | .235*** | .726*** |

*Note.* Attachment avoidance and anxiety were assessed at background. All other variables were assessed at the daily level. Correlations with daily variables are with the between-person aggregates across the entire 21-day period of the study.

† < .10, *** *p* < .001

# **Table S3**

## **Bivariate correlations among all variables in Sample 3**

|  | 1 | 2 | 3 | 4 | 5 | 6 |
| --- | --- | --- | --- | --- | --- | --- |
| 1. Attachment Avoidance | — |  |  |  |  |  |
| 2. Attachment Anxiety | .267*** | — |  |  |  |  |
| 3. Novelty and Excitement | -.084 | -.066 | — |  |  |  |
| 4. Familiarity and Comfort | -.354*** | -.237*** | .399*** | — |  |  |
| 5. Relational Reward | -.421*** | -.105 | .330*** | .792*** | — |  |
| 6. Relational Threat | .343*** | .411*** | .049 | .373*** | -.297*** | — |
| 7. Relationship Satisfaction | -.416*** | -.273*** | .336*** | .803*** | .776*** | -.468*** |

*Note.* Attachment avoidance and anxiety were assessed at background. All other variables were assessed at the daily level. Correlations with daily variables are with the between-person aggregates across the entire 21-day period of the study.

*** *p* < .001

| **Table S4**  **Meta-Analyzed Effects of Attachment Insecurity and Shared Experiences Predicting Daily Relationship Satisfaction** | | | | | |
| --- | --- | --- | --- | --- | --- |
|  | DV: Relationship Satisfaction | | | | |
|  | *Estimate* | *SE* | *z* | *p* | *95% CI* |
| Avoidance | -0.13 | 0.03 | -5.02 | <.001 | [-0.18, -0.08] |
| Anxiety | -0.09 | 0.03 | -2.72 | .007 | [-0.15, -0.02] |
| Novel and Exciting (Within-Person) | 0.13 | 0.02 | 5.70 | <.001 | [0.08, 0.17] |
| Novel and Exciting (Between-Person) | 0.09 | 0.03 | 3.26 | .001 | [0.03, 0.14] |
| Comfortable and Familiar (Within-Person) | 0.30 | 0.02 | 15.02 | <.001 | [0.26, 0.34] |
| Comfortable and Familiar (Between-Person) | 0.69 | 0.02 | 30.79 | <.001 | [0.65, 0.74] |
| Avoidance x Novel and Exciting (Within-Person) | 0.02 | 0.01 | 3.99 | <.001 | [0.01, 0.04] |
| Avoidance x Novel and Exciting (Between-Person) | 0.01 | 0.04 | 0.21 | .834 | [-0.06, 0.08] |
| Avoidance x Comfortable and Familiar (Within-Person) | 0.01 | 0.02 | 0.38 | .702 | [-0.03, 0.05] |
| Avoidance x Comfortable and Familiar (Between-Person) | 0.10 | 0.07 | 1.53 | .126 | [-0.03, 0.23] |
| Anxiety x Novel and Exciting (Within-Person) | 0.02 | 0.01 | 1.91 | .055 | [-0.0004, 0.04] |
| Anxiety x Novel and Exciting (Between-Person) | 0.04 | 0.04 | 1.00 | .317 | [-0.04, 0.12] |
| Anxiety x Comfortable and Familiar (Within-Person) | 0.03 | 0.01 | 2.16 | .031 | [0.03, 0.06] |
| Anxiety x Comfortable and Familiar (Between-Person) | 0.09 | 0.03 | 3.55 | <.001 | [0.04, 0.15] |

*Note.* Estimates are derived from a meta-analysis of three samples. For each sample, a multilevel model was conducted in which all predictors in the table were simultaneously entered to predict the outcome.

| **Table S5** | | | | | | | | | | |
| --- | --- | --- | --- | --- | --- | --- | --- | --- | --- | --- |
| ***Meta-Analyzed Effects of Attachment Insecurity and Shared Experiences Predicting Daily Relational Reward and Threat (A Path)*** | | | | | | | | | | |
|  | Relational Reward | | | | | Relational Threat | | | | |
|  | *Estimate* | *SE* | *z* | *p* | *95% CI* | *Estimate* | *SE* | *z* | *p* | *95% CI* |
| Avoidance (Between-Person) | -0.26 | 0.04 | -6.47 | <.001 | [-0.34, -0.18] | 0.15 | 0.08 | 1.86 | .06 | [-0.01, 0.31] |
| Anxiety (Between-Person) | 0.06 | 0.13 | 0.47 | .641 | [-0.19, 0.31] | 0.24 | 0.07 | 3.28 | .001 | [0.10, 0.39] |
| Novel and Exciting (Within-Person) | 0.16 | 0.05 | 3.06 | .002 | [0.06, 0.27] | 0.05 | 0.02 | 2.89 | .004 | [0.01, 0.08] |
| Novel and Exciting (Between-Person) | 0.12 | 0.06 | 1.95 | .051 | [-0.001, 0.24] | 0.25 | 0.04 | 6.17 | <.001 | [0.17, 0.33] |
| Comfortable and Familiar (Within-Person) | 0.34 | 0.02 | 14.65 | <.001 | [0.29, 0.38] | -0.15 | 0.02 | -7.17 | <.001 | [-0.19, -0.11] |
| Comfortable and Familiar (Between-Person) | 0.69 | 0.03 | 19.95 | <.001 | [0.62, 0.76] | -0.29 | 0.07 | -4.26 | <.001 | [-0.43, -0.16] |
| Relational Reward (Within-Person) | 0.06 | 0.03 | 2.33 | .020 | [0.01, 0.11] | 0.04 | 0.03 | 1.38 | .168 | [-0.02, 0.09] |
| Relational Reward (Between-Person) | 0.04 | 0.08 | 0.43 | .666 | [-0.13, 0.20] | 0.11 | 0.04 | 2.56 | 0.011 | [0.02, 0.19] |
| Relational Threat (Within-Person) | 0.01 | 0.02 | 0.54 | .590 | [-0.03, 0.05] | 0.04 | 0.01 | 4.11 | <.001 | [0.02, 0.06] |
| Relational Threat (Between-Person) | 0.05 | 0.11 | 0.44 | 0.663 | [-0.17, 0.26] | 0.02 | 0.13 | 0.14 | 0.891 | [-0.24, 0.28] |
| Avoidance (Between-Person) x Novel and Exciting (Within-Person) | 0.01 | 0.04 | 0.26 | .798 | [-0.06, 0.08] | -0.01 | 0.04 | -0.16 | .871 | [-0.08, 0.07] |
| Avoidance (Between-Person) x Novel and Exciting (Between-Person) | -0.003 | 0.04 | -0.07 | 0.947 | [-0.08, 0.08] | 0.05 | 0.10 | 0.55 | 0.582 | [-0.13, 0.24] |
| Avoidance (Between-Person) x Comfortable and Familiar (Within-Person) | 0.02 | 0.01 | 1.60 | .110 | [-0.003, 0.03] | 0.01 | 0.01 | 1.16 | .244 | [-0.01, 0.03] |
| Avoidance (Between-Person) x Comfortable and Familiar (Between-Person) | 0.13 | 0.14 | 0.92 | 0.356 | [-0.14, 0.39] | -0.003 | 0.05 | -0.06 | 0.951 | [-0.11, 0.10] |

*Note.* Estimates are derived from a meta-analysis of three samples. For each sample, a multilevel model was conducted in which all predictors in the table were simultaneously entered to predict the outcome.

| **Table S6** | | | | | |
| --- | --- | --- | --- | --- | --- |
| ***Meta-Analyzed Effects of Attachment Insecurity, Shared Experiences and Relational Reward and Threat Predicting Daily Relationship Satisfaction (B Paths and Direct Effects)*** | | | | | |
|  | DV: Relationship Satisfaction | | | | |
|  | *Estimate* | *SE* | *z* | *p* | *95% CI* |
| Avoidance (Between-Person) | -0.02 | .03 | -0.67 | .501 | [-0.08, 0.04] |
| Anxiety (Between-Person) | -0.03 | .03 | -1.06 | .287 | [-0.09, 0.03] |
| Novel and Exciting (Within-Person) | 0.07 | .01 | 10.45 | <.001 | [0.06, 0.08] |
| Novel and Exciting (Between-Person) | 0.09 | .03 | 3.03 | .003 | [0.03, 0.15] |
| Comfortable and Familiar (Within-Person) | 0.16 | .01 | 10.55 | <.001 | [0.13, 0.19] |
| Comfortable and Familiar (Between-Person) | 0.32 | .05 | 6.25 | <.001 | [0.22, 0.43] |
| Relational Reward (Within-Person) | 0.22 | .03 | 8.42 | <.001 | [0.17, 0.27] |
| Relational Reward (Between-Person) | 0.34 | .05 | 6.42 | <.001 | [0.23, 0.44] |
| Relational Threat (Within-Person) | -0.10 | .02 | -5.01 | <.001 | [-0.14, -0.06] |
| Relational Threat (Between-Person) | -0.12 | .06 | -2.00 | .046 | [-0.24, -0.002] |
| Avoidance (Between-Person) x Novel and Exciting (Within-Person) | 0.02 | .01 | 3.07 | .002 | [0.01, 0.03] |
| Avoidance (Between-Person) x Novel and Exciting (Between-Person) | 0.06 | .03 | 1.99 | .047 | [0.001, 0.12] |
| Avoidance (Between-Person) x Comfortable and Familiar (Within-Person) | -0.002 | .01 | -0.24 | .807 | [-0.01, 0.01] |
| Avoidance (Between-Person) x Comfortable and Familiar (Between-Person) | 0.03 | .08 | 0.35 | .725 | [-0.13, 0.19] |
| Anxiety (Between-Person) x Novel and Exciting (Within-Person) | -0.002 | .01 | -0.29 | .770 | [-0.02, 0.01] |
| Anxiety (Between-Person) x Novel and Exciting (Between-Person) | -0.01 | .03 | -0.50 | .620 | [-0.07, 0.04] |
| Anxiety (Between-Person) x Comfortable and Familiar (Within-Person) | 0.02 | .01 | 3.25 | .001 | [0.01, 0.04] |
| Anxiety (Between-Person) x Comfortable and Familiar (Between-Person) | 0.03 | .02 | 0.85 | .394 | [-0.03, 0.08] |
| Avoidance (Between-Person) x Relational Reward (Within-Person) | -0.02 | .01 | -1.31 | .190 | [-0.05, 0.01] |
| Avoidance (Between-Person) x Relational Reward (Between-Person) | -0.04 | .03 | -1.15 | .249 | [-0.11, 0.03] |
| Avoidance (Between-Person) x Relational Threat (Within-Person) | -0.003 | .01 | -0.40 | .690 | [-0.02, 0.01] |
| Avoidance (Between-Person) x Relational Threat (Between-Person) | -0.04 | .05 | -0.81 | .418 | [-0.14, 0.06] |
| Anxiety (Between-Person) x Relational Reward (Within-Person) | 0.04 | .02 | 1.91 | .056 | [-0.001, 0.09] |
| Anxiety (Between-Person) Relational Reward (Between-Person) | .02 | .03 | 0.57 | .570 | [-0.05, 0.09] |
| Anxiety (Between-Person) Relational Threat (Within-Person) | .003 | .01 | 0.485 | .628 | [-0.01, 0.02] |
| Anxiety (Between-Person) Relational Threat (Between-Person) | .09 | .04 | 2.33 | .020 | [0.01, 0.17] |

*Note.* Estimates are derived from a meta-analysis of three samples. For each sample, a multilevel model was conducted in which all predictors in the table were simultaneously entered to predict the outcome.

| **Table S7** | | | | | |
| --- | --- | --- | --- | --- | --- |
| ***Meta-Analyzed Effects of Actor and Partner Attachment Insecurity and Actor Reports of Shared Experiences Predicting Daily Relationship Satisfaction*** | | | | | |
|  | DV: Relationship Satisfaction | | | | |
|  | *Estimate* | *SE* | *z* | *p* | *95% CI* |
| Actor Avoidance (Between-Person) | -0.12 | 0.03 | -4.59 | <.001 | [-0.17, -0.07] |
| Partner Avoidance (Between-Person) | -0.06 | 0.05 | -1.17 | .243 | [-0.16, 0.04] |
| Actor Anxiety (Between-Person) | -0.09 | 0.05 | -1.89 | .059 | [-0.17, 0.003] |
| Partner Anxiety (Between-Person) | -0.09 | 0.05 | -1.81 | .071 | [-0.18, 0.01] |
| Actor Novel and Exciting (Within-Person) | 0.13 | 0.02 | 6.11 | <.001 | [0.09, 0.17] |
| Actor Comfortable and Familiar (Within-Person) | 0.30 | 0.02 | 15.54 | <.001 | [0.26, 0.34] |
| Actor Avoidance (Between-Person) x Novel and Exciting (Within-Person) | 0.04 | 0.01 | 3.47 | <.001 | [0.01, 0.04] |
| Partner Avoidance (Between-Person) x Novel and Exciting (Within-Person) | 0.02 | 0.01 | 2.34 | .019 | [0.003, 0.03] |
| Actor Avoidance (Between-Person) x Comfortable and Familiar (Within-Person) | -0.003 | 0.02 | -0.14 | .890 | [-0.05, 0.04] |
| Partner Avoidance (Between-Person) x Comfortable and Familiar (Within-Person) | -0.01 | 0.02 | -0.53 | .596 | [-0.04, 0.02] |
| Actor Anxiety (Between-Person) x Novel and Exciting (Within-Person) | 0.01 | 0.01 | 1.23 | .220 | [-0.01, 0.04] |
| Partner Anxiety (Between-Person) x Novel and Exciting (Within-Person) | -0.005 | 0.01 | -0.65 | .514 | [-0.02, 0.01] |
| Actor Anxiety (Between-Person) x Comfortable and Familiar (Within-Person) | 0.04 | 0.02 | 2.11 | .035 | [0.01, 0.07] |
| Partner Anxiety (Between-Person) x Comfortable and Familiar (Within-Person) | 0.04 | 0.01 | 6.59 | <.001 | [0.03, 0.05] |

*Note.* Estimates are derived from a meta-analysis of three samples. For each sample, a multilevel model was conducted in which all predictors in the table were simultaneously entered to predict the outcome.

| **Table S8**  **Meta-Analyzed Effects of Attachment Insecurity and Shared Experiences Predicting Daily Relationship Satisfaction Controlling for Previous Day Outcome (Yesterday’s Relationship Satisfaction)** | | | | | |
| --- | --- | --- | --- | --- | --- |
|  | DV: Relationship Satisfaction | | | | |
|  | *Estimate* | *SE* | *z* | *p* | *95% CI* |
| Previous Day Relationship Satisfaction | 0.09 | 0.01 | 6.65 | <.001 | [0.06, 0.11] |
| Avoidance | -0.13 | 0.03 | -4.95 | <.001 | [-0.18, -0.08] |
| Anxiety | -0.08 | 0.04 | -2.00 | .046 | [-0.15, -0.001] |
| Novel and Exciting (Within-Person) | 0.13 | 0.02 | 6.52 | <.001 | [0.09, 0.17] |
| Novel and Exciting (Between-Person) | 0.09 | 0.03 | 3.24 | .001 | [0.03, 0.14] |
| Comfortable and Familiar (Within-Person) | 0.30 | 0.02 | 14.81 | <.001 | [0.26, 0.34] |
| Comfortable and Familiar (Between-Person) | 0.72 | 0.05 | 14.11 | <.001 | [0.62, 0.82] |
| Avoidance x Novel and Exciting (Within-Person) | 0.02 | 0.01 | 2.23 | .026 | [0.003, 0.04] |
| Avoidance x Novel and Exciting (Between-Person) | 0.01 | 0.04 | 0.16 | .875 | [-0.07, 0.08] |
| Avoidance x Comfortable and Familiar (Within-Person) | 0.01 | 0.02 | 0.42 | .678 | [-0.03, 0.05] |
| Avoidance x Comfortable and Familiar (Between-Person) | 0.10 | 0.06 | 1.73 | .083 | [-0.01, 0.22] |
| Anxiety x Novel and Exciting (Within-Person) | 0.02 | 0.01 | 3.02 | .003 | [0.01, 0.04] |
| Anxiety x Novel and Exciting (Between-Person) | 0.04 | 0.04 | 0.95 | .341 | [-0.04, 0.12] |
| Anxiety x Comfortable and Familiar (Within-Person) | 0.04 | 0.02 | 2.21 | .027 | [0.004, 0.07] |
| Anxiety x Comfortable and Familiar (Between-Person) | 0.08 | 0.03 | 3.14 | .002 | [0.03, 0.13] |

*Note.* Estimates are derived from a meta-analysis of three samples. For each sample, a multilevel model was conducted in which all predictors in the table were simultaneously entered to predict the outcome.

| **Table S9** | | | | | | | | | | |
| --- | --- | --- | --- | --- | --- | --- | --- | --- | --- | --- |
| ***Effects of Attachment Insecurity and Shared Experiences Predicting Daily Positive and Negative Affect (A Path; Sample 3)*** | | | | | | | | | | |
| *Predictors* | *Criterion* | | | | | | | | | |
|  | Positive Affect | | | | | Negative Affect | | | | |
|  | *b* | *SE* | *t* | *p* | 95% *CI* | *b* | *SE* | *t* | *p* | 95% *CI* |
| Attachment Avoidance (Between-Person) | -0.01 | 0.06 | -0.21 | .836 | [-0.12, 0.10] | 0.03 | 0.07 | 0.39 | .694 | [-0.10, 0.16] |
| Attachment Anxiety (Between-Person) | -0.13 | 0.04 | -3.66 | <.001 | [-0.21, -0.06] | 0.28 | 0.04 | 6.23 | <.001 | [0.19, 0.36] |
| Novelty and Excitement (Within-Person) | 0.22 | 0.01 | 16.33 | <.001 | [-0.21, -0.06] | -0.02 | 0.01 | -1.54 | .123 | [-0.05, 0.01] |
| Novelty and Excitement (Between-Person) | 0.18 | 0.04 | 4.98 | <.001 | [0.11, 0.25] | -0.06 | 0.04 | -1.29 | .198 | [-0.14, 0.03] |
| Familiarity and Comfort (Within-Person) | 0.29 | 0.02 | 19.62 | <.001 | [0.27, 0.32] | -0.21 | 0.02 | -13.13 | <.001 | [-0.24, -0.17] |
| Familiarity and Comfort (Between-Person) | 0.53 | 0.06 | 9.29 | <.001 | [0.42, 0.95] | -0.20 | 0.07 | -2.85 | .005 | [-0.33, -0.06] |
| Attachment Avoidance (Between-Person) x Novelty and Excitement (Within-Person) | 0.01 | 0.02 | 0.69 | .490 | [-0.02, 0.04] | 0.09 | 0.02 | 5.76 | <.001 | [0.06, 0.12] |
| Attachment Avoidance (Between-Person) x Novelty and Excitement (Between-Person) | -0.01 | 0.04 | -0.22 | .824 | [-0.09, 0.07] | 0.02 | 0.05 | 0.35 | .729 | [-0.08, 0.12] |
| Attachment Avoidance (Between-Person) x Familiarity and Comfort (Within-Person) | 0.002 | 0.02 | 0.15 | .880 | [-0.03, 0.03] | 0.03 | 0.02 | 2.12 | .035 | [0.002, 0.07] |
| Attachment Avoidance (Between-Person) x Familiarity and Comfort (Between-Person) | 0.04 | 0.05 | 0.77 | .440 | [-0.06, 0.14] | -0.13 | 0.06 | -2.21 | .028 | [-0.25, -0.01] |
| Attachment Anxiety (Between-Person) x Novelty and Excitement (Within-Person) | 0.02 | 0.01 | 2.14 | .032 | [0.002, 0.04] | -0.04 | 0.01 | -3.31 | <.001 | [-0.06, -0.02] |
| Attachment Anxiety (Between-Person) x Novelty and Excitement (Between-Person) | -0.002 | 0.03 | -0.08 | .936 | [-0.06, 0.05] | -0.001 | 0.01 | -3.31 | <.001 | [-0.07, 0.07] |
| Attachment Anxiety (Between-Person) x Familiarity and Comfort (Within-Person) | 0.02 | 0.01 | 1.80 | .071 | [-0.002, 0.04] | -0.03 | 0.01 | -2.25 | .024 | [-0.05, -0.004] |
| Attachment Anxiety (Between-Person) x Familiarity and Comfort (Between-Person) | -0.02 | 0.04 | -0.37 | .709 | [-0.10, 0.07] | -0.03 | 0.05 | -0.50 | .620 | [-0.13, 0.08] |

*Note.* Estimates are derived from a multilevel model in which all predictors in the table were simultaneously entered to predict the outcome. *b* coefficients reflect unstandardized multilevel modeling coefficients.

| **Table S10** | | | | | |
| --- | --- | --- | --- | --- | --- |
| ***Direct effect – B Path (Sample 3)*** | | | | | |
|  | DV: Relationship Satisfaction | | | | |
|  | *b* | *SE* | *t* | *p* | *95% CI* |
| Avoidance (Between-Person) | -0.11 | 0.04 | -2.94 | .004 | [-0.19, -0.04] |
| Anxiety (Between-Person) | -0.03 | 0.03 | -0.96 | .337 | [-0.08, 0.03] |
| Novel and Exciting (Within-Person) | 0.05 | 0.01 | 4.54 | <.001 | [0.03, 0.07] |
| Novel and Exciting (Between-Person) | 0.02 | 0.03 | 0.64 | .522 | [-0.04, 0.07] |
| Comfortable and Familiar (Within-Person) | 0.26 | 0.01 | 22.11 | <.001 | [0.23, 0.28] |
| Comfortable and Familiar (Between-Person) | 0.49 | 0.05 | 10.34 | <.001 | [0.40, 0.59] |
| Positive Affect (Within-Person) | 0.14 | 0.01 | 11.54 | <.001 | [0.12, 0.17] |
| Positive Affect (Between-Person) | 0.17 | 0.05 | 3.18 | .002 | [0.06, 0.27] |
| Negative Affect (Within-Person) | -0.06 | 0.01 | -4.73 | <.001 | [-0.09, -0.03] |
| Negative Affect (Between-Person) | 0.01 | 0.05 | 0.10 | .918 | [-0.08, 0.09] |
| Avoidance (Between-Person) x Novel and Exciting (Within-Person) | 0.01 | 0.01 | 0.93 | .354 | [-0.02, 0.03] |
| Avoidance (Between-Person) x Novel and Exciting (Between-Person) | 0.02 | 0.03 | 0.62 | .533 | [-0.04, 0.08] |
| Avoidance (Between-Person) x Comfortable and Familiar (Within-Person) | -0.01 | 0.01 | -0.44 | .662 | [-0.03, 0.02] |
| Avoidance (Between-Person) x Comfortable and Familiar (Between-Person) | 0.15 | 0.05 | 3.07 | .002 | [0.05, 0.25] |
| Anxiety (Between-Person) x Novel and Exciting (Within-Person) | -0.001 | 0.01 | -0.13 | .894 | [-0.02, 0.02] |
| Anxiety (Between-Person) x Novel and Exciting (Between-Person) | -0.001 | 0.02 | -0.05 | .958 | [-0.04, 0.04] |
| Anxiety (Between-Person) x Comfortable and Familiar (Within-Person) | 0.04 | 0.01 | 4.10 | <.001 | [0.02, 0.06] |
| Anxiety (Between-Person) x Comfortable and Familiar (Between-Person) | 0.09 | 0.04 | 2.24 | .026 | [0.01, 0.16] |
| Avoidance (Between-Person) x Positive Affect (Within-Person) | 0.05 | 0.01 | 3.63 | <.001 | [0.02, 0.08] |
| Avoidance (Between-Person) x Positive Affect (Between-Person) | -0.03 | 0.06 | -0.47 | .641 | [-0.14, 0.09] |
| Avoidance (Between-Person) x Negative Affect (Within-Person) | 0.04 | 0.01 | 3.07 | .002 | [0.01, 0.06] |
| Avoidance (Between-Person) x Negative Affect (Between-Person) | 0.02 | 0.05 | 0.39 | .697 | [-0.08, .11] |
| Anxiety (Between-Person) x Positive Affect (Within-Person) | -0.002 | 0.01 | -0.22 | .828 | [-0.02, 0.02] |
| Anxiety (Between-Person) Positive Affect (Between-Person) | -0.06 | 0.04 | -1.49 | .139 | [-0.15, 0.02] |
| Anxiety (Between-Person) Negative Affect (Within-Person) | -0.01 | 0.01 | -1.19 | .236 | [-0.03, 0.01] |
| Anxiety (Between-Person) Negative Affect (Between-Person) | -0.001 | 0.03 | -0.03 | .975 | [-0.06, 0.06] |

*Note.* Estimates are derived from a multilevel model in which all predictors in the table were simultaneously entered to predict the outcome. *b* coefficients reflect unstandardized multilevel modeling coefficients.

| **Table S11** | | | | | |
| --- | --- | --- | --- | --- | --- |
| *Study 1 - The Effects of Attachment Insecurity and Shared Experiences Predicting Daily Relationship Satisfaction* | | | | | |
| *Predictors* | *Criterion* | | | | |
|  | Relationship Satisfaction | | | | |
|  | *b* | *SE* | *t* | *p* | 95% CI |
| Attachment Avoidance (Between-Person) | -0.09 | 0.04 | -2.24 | .026 | [-0.17, -0.01] |
| Attachment Anxiety (Between-Person) | -0.02 | 0.03 | -0.638 | .524 | [-0.08, 0.04] |
| Novelty and Excitement (Within-Person) | 0.13 | 0.01 | 12.57 | <.001 | [0.11, 0.15] |
| Novelty and Excitement (Between-Person) | 0.06 | 0.02 | 2.72 | .007 | [0.02, 0.11] |
| Familiarity and Comfort (Within-Person) | 0.33 | 0.01 | 26.04 | <.001 | [0.31, 0.36] |
| Familiarity and Comfort (Between-Person) | 0.67 | 0.04 | 17.78 | <.001 | [0.59, 0.74] |
| Attachment Avoidance (Between-Person) x Novelty and Excitement (Within-Person) | 0.05 | 0.01 | 3.41 | <.001 | [0.02, 0.07] |
| Attachment Avoidance (Between-Person) x Novelty and Excitement (Between-Person) | 0.03 | 0.03 | 1.16 | .248 | [-0.02, 0.09] |
| Attachment Avoidance (Between-Person) x Familiarity and Comfort (Within-Person) | -0.04 | 0.02 | -2.24 | .025 | [-0.07, -0.004] |
| Attachment Avoidance (Between-Person) x Familiarity and Comfort (Between-Person) | -0.02 | 0.03 | -0.640 | .523 | [-0.09, 0.05] |
| Attachment Anxiety (Between-Person) x Novelty and Excitement (Within-Person) | 0.01 | 0.01 | 1.37 | .1712 | [-0.01, 0.03] |
| Attachment Anxiety (Between-Person) x Novelty and Excitement (Between-Person) | 0.03 | 0.02 | 1.44 | .153 | [-0.01, 0.07] |
| Attachment Anxiety (Between-Person) x Familiarity and Comfort (Within-Person) | 0.04 | 0.01 | 3.50 | <.001 | [0.02, 0.06] |
| Attachment Anxiety (Between-Person) x Familiarity and Comfort (Between-Person) | 0.05 | 0.03 | 1.70 | .090 | [-0.01, 0.11] |

*Note.* Estimates are derived from a multilevel model in which all predictors in the table were simultaneously entered to predict the outcome. *b* coefficients reflect unstandardized multilevel modeling coefficients.

| **Table S12** | | | | | | | | | | |
| --- | --- | --- | --- | --- | --- | --- | --- | --- | --- | --- |
| ***Study 1 Effects of Attachment Insecurity and Shared Experiences Predicting Relational Reward and Threat (A Path)*** | | | | | | | | | | |
| *Predictors* | *Criterion* | | | | | | | | | |
|  | Relational Reward | | | | | Relational Threat | | | | |
|  | *b* | *SE* | *t* | *p* | 95% CI | *b* | *SE* | *t* | *p* | 95% CI |
| Attachment Avoidance (Between-Person) | -0.16 | 0.03 | -4.95 | <.001 | [0.23, -0.10] | 0.05 | 0.05 | 1.180 | .239 | [-0.04, 0.15] |
| Attachment Anxiety (Between-Person) | -0.03 | 0.02 | -1.13 | .262 | [-0.07, 0.02] | 0.09 | 0.03 | 2.84 | .005 | [0.03, 0.16] |
| Novelty and Excitement (Within-Person) | 0.10 | 0.01 | 15.73 | <.001 | [0.09, 0.11] | 0.04 | 0.01 | 4.56 | <.001 | [0.02, 0.06] |
| Novelty and Excitement (Between-Person) | 0.06 | 0.02 | 3.02 | .003 | [0.02, 0.10] | 0.14 | 0.03 | 4.49 | <.001 | [0.08, 0.20] |
| Familiarity and Comfort (Within-Person) | 0.20 | 0.01 | 26.43 | <.001 | [0.19, 0.22] | -0.10 | 0.01 | -9.14 | <.001 | [-0.12, -0.08] |
| Familiarity and Comfort (Between-Person) | 0.36 | 0.03 | 11.18 | <.001 | [0.29, 0.42] | -0.28 | 0.05 | -5.74 | <.001 | [-0.37, -0.18] |
| Attachment Avoidance (Between-Person) x Novelty and Excitement (Within-Person) | 0.05 | 0.01 | 6.28 | <.001 | [0.04, 0.07] | 0.01 | 0.01 | 0.77 | .440 | [-0.01, 0.03] |
| Attachment Avoidance (Between-Person) x Novelty and Excitement (Between-Person) | 0.05 | 0.02 | 1.96 | .051 | [-0.0002, 0.09] | 0.04 | 0.03 | 1.25 | .212 | [-0.02, 0.11] |
| Attachment Avoidance (Between-Person) x Familiarity and Comfort (Within-Person) | -0.01 | 0.01 | -0.71 | .476 | [-0.03, 0.01] | 0.03 | 0.01 | 2.48 | .013 | [0.01, 0.06] |
| Attachment Avoidance (Between-Person) x Familiarity and Comfort (Between-Person) | -0.03 | 0.03 | -1.00 | .321 | [-0.08, 0.03] | -0.05 | 0.04 | -1.23 | .221 | [-0.13, 0.03] |
| Attachment Anxiety (Between-Person) x Novelty and Excitement (Within-Person) | 0.02 | 0.01 | 3.39 | <.001 | [0.01, 0.03] | 0.02 | 0.01 | 2.30 | .022 | [0.003, 0.04] |
| Attachment Anxiety (Between-Person) x Novelty and Excitement (Between-Person) | 0.01 | 0.02 | 0.64 | .526 | [-0.02, 0.04] | 0.06 | 0.02 | 2.37 | .019 | [0.01, 0.10] |
| Attachment Anxiety (Between-Person) x Familiarity and Comfort (Within-Person) | 0.002 | 0.01 | 0.40 | .689 | [-0.01, 0.02] | 0.01 | 0.01 | 0.68 | .499 | [-0.01, 0.03] |
| Attachment Anxiety (Between-Person) x Familiarity and Comfort (Between-Person) | 0.10 | .02 | 4.18 | <.001 | [0.05, 0.15] | 0.03 | 0.03 | 0.760 | .448 | [-0.04, 0.09] |

*Note.* Estimates are derived from a multilevel model in which all predictors in the table were simultaneously entered to predict the outcome. *b* coefficients reflect unstandardized multilevel modeling coefficients.

| **Table S13** | | | | | |
| --- | --- | --- | --- | --- | --- |
| ***Study 1 Effects of Attachment Insecurity, Shared Experiences and Relational Reward and Threat Predicting Daily Relationship Satisfaction (B Paths and Direct Effects)*** | | | | | |
|  | DV: Relationship Satisfaction | | | | |
|  | *Estimate* | *SE* | *t* | *p* | *95% CI* |
| Avoidance (Between-Person) | 0.01 | 0.04 | 0.36 | .719 | [-0.06, 0.09] |
| Anxiety (Between-Person) | 0.0004 | 0.02 | -0.002 | .999 | [-0.05, 0.05] |
| Novel and Exciting (Within-Person) | 0.08 | 0.01 | 7.92 | <.001 | [0.06, 0.10] |
| Novel and Exciting (Between-Person) | 0.04 | 0.02 | 1.74 | .084 | [0.005, 0.08] |
| Comfortable and Familiar (Within-Person) | 0.20 | 0.01 | 15.46 | <.001 | [0.17, 0.22] |
| Comfortable and Familiar (Between-Person) | 0.37 | 0.04 | 8.50 | <.001 | [0.29, 0.46] |
| Relational Reward (Within-Person) | 0.63 | 0.02 | 25.75 | <.001 | [0.58, 0.68] |
| Relational Reward (Between-Person) | 0.66 | 0.07 | 8.94 | <.001 | [0.51, 0.81] |
| Relational Threat (Within-Person) | -0.16 | 0.02 | -8.70 | <.001 | [-0.20, -0.13] |
| Relational Threat (Between-Person) | -0.07 | 0.05 | -1.38 | <.001 | [-0.20, -0.13] |
| Avoidance (Between-Person) x Novel and Exciting (Within-Person) | 0.03 | 0.01 | -8.70 | <.001 | [-0.20, -0.13] |
| Avoidance (Between-Person) x Novel and Exciting (Between-Person) | 0.03 | 0.03 | 1.01 | .312 | [-0.02, 0.08] |
| Avoidance (Between-Person) x Comfortable and Familiar (Within-Person) | -0.01 | 0.02 | -0.53 | .596 | [-0.04, 0.02] |
| Avoidance (Between-Person) x Comfortable and Familiar (Between-Person) | -0.06 | 0.02 | -8.70 | <.001 | [-0.20, -0.13] |
| Anxiety (Between-Person) x Novel and Exciting (Within-Person) | -0.001 | 0.01 | -0.14 | .887 | [-0.02, 0.02] |
| Anxiety (Between-Person) x Novel and Exciting (Between-Person) | 0.03 | 0.02 | 0.150 | .881 | [-0.03, 0.04] |
| Anxiety (Between-Person) x Comfortable and Familiar (Within-Person) | 0.03 | 0.01 | 2.95 | .003 | [0.01, 0.06] |
| Anxiety (Between-Person) x Comfortable and Familiar (Between-Person) | 0.03 | 0.04 | 0.78 | .438 | [-0.05, 0.11] |
| Avoidance (Between-Person) x Relational Reward (Within-Person) | -0.10 | 0.03 | -3.38 | <.001 | [-0.15, -0.04] |
| Avoidance (Between-Person) x Relational Reward (Between-Person) | 0.01 | 0.06 | 0.14 | .886 | [-0.11, 0.13] |
| Avoidance (Between-Person) x Relational Threat (Within-Person) | -0.02 | 0.02 | -0.96 | .336 | [-0.06, 0.02] |
| Avoidance (Between-Person) x Relational Threat (Between-Person) | -0.09 | 0.05 | -2.00 | .047 | [-0.18, -0.001] |
| Anxiety (Between-Person) x Relational Reward (Within-Person) | 0.05 | 0.02 | 2.12 | .034 | [0.004, 0.10] |
| Anxiety (Between-Person) Relational Reward (Between-Person) | -0.01 | 0.05 | -0.11 | .913 | [-0.11, 0.10] |
| Anxiety (Between-Person) Relational Threat (Within-Person) | 0.01 | 0.01 | 0.42 | .674 | [-0.03, 0.04] |
| Anxiety (Between-Person) Relational Threat (Between-Person) | 0.11 | 0.04 | 3.01 | .003 | [0.04, 0.18] |

*Note.* Estimates are derived from a multilevel model in which all predictors in the table were simultaneously entered to predict the outcome. *b* coefficients reflect unstandardized multilevel modeling coefficients.

| **Table S14** | | | | | |
| --- | --- | --- | --- | --- | --- |
| ***Study 2 Effects of Attachment Insecurity and Shared Experiences Predicting Daily Relationship Satisfaction*** | | | | | |
| *Predictors* | *Criterion* | | | | |
|  | Relationship Satisfaction | | | | |
|  | *b* | *SE* | *t* | *p* | 95% CI |
| Attachment Avoidance (Between-Person) | -0.10 | 0.03 | -3.12 | .002 | [-0.17, -0.04] |
| Attachment Anxiety (Between-Person) | -0.06 | 0.02 | -3.06 | .002 | [-0.10, -0.02] |
| Novelty and Excitement (Within-Person) | 0.14 | 0.01 | 16.35 | <.001 | [0.12, 0.16] |
| Novelty and Excitement (Between-Person) | 0.03 | 0.03 | 1.07 | .287 | [-0.02, 0.08] |
| Familiarity and Comfort (Within-Person) | 0.40 | 0.01 | 33.94 | <.001 | [0.38, 0.43] |
| Familiarity and Comfort (Between-Person) | 0.60 | 0.04 | 14.87 | <.001 | [0.52, 0.68] |
| Attachment Avoidance (Between-Person) x Novelty and Excitement (Within-Person) | 0.01 | 0.01 | 1.59 | .113 | [-0.003, 0.03] |
| Attachment Avoidance (Between-Person) x Novelty and Excitement (Between-Person) | -0.04 | 0.03 | -1.37 | .171 | [-0.09, 0.02] |
| Attachment Avoidance (Between-Person) x Familiarity and Comfort (Within-Person) | 0.05 | 0.01 | 4.21 | <.001 | [0.03, 0.07] |
| Attachment Avoidance (Between-Person) x Familiarity and Comfort (Between-Person) | 0.11 | 0.04 | 3.25 | .001 | [0.04, 0.18] |
| Attachment Anxiety (Between-Person) x Novelty and Excitement (Within-Person) | 0.02 | 0.01 | 3.97 | <.001 | [0.01, 0.03] |
| Attachment Anxiety (Between-Person) x Novelty and Excitement (Between-Person) | 0.03 | 0.01 | 2.08 | .038 | [0.002, 0.06] |
| Attachment Anxiety (Between-Person) x Familiarity and Comfort (Within-Person) | 0.003 | 0.01 | 0.40 | .687 | [-0.01, 0.02] |
| Attachment Anxiety (Between-Person) x Familiarity and Comfort (Between-Person) | 0.06 | 0.02 | 2.58 | .010 | [0.01, 0.11] |

*Note.* Estimates are derived from a multilevel model in which all predictors in the table were simultaneously entered to predict the outcome. *b* coefficients reflect unstandardized multilevel modeling coefficients.

| **Table S15** | | | | | |
| --- | --- | --- | --- | --- | --- |
| ***Study 3 - The Effects of Attachment Insecurity and Shared Experiences Predicting Daily Relationship Satisfaction*** | | | | | |
| *Predictors* | *Criterion* | | | | |
|  | Relationship Satisfaction | | | | |
|  | *b* | *SE* | *t* | *p* | 95% CI |
| Attachment Avoidance (Between-Person) | -0.11 | 0.04 | -2.96 | .003 | [-0.19, -0.04] |
| Attachment Anxiety (Between-Person) | -0.04 | 0.03 | -1.69 | .092 | [-0.09, 0.01] |
| Novelty and Excitement (Within-Person) | 0.08 | 0.01 | 8.18 | <.001 | [0.06, 0.10] |
| Novelty and Excitement (Between-Person) | 0.05 | 0.03 | 1.91 | .057 | [-0.001, 0.10] |
| Familiarity and Comfort (Within-Person) | 0.31 | 0.01 | 26.96 | <.001 | [0.29, 0.33] |
| Familiarity and Comfort (Between-Person) | 0.58 | 0.04 | 14.09 | <.001 | [0.50, 0.66] |
| Attachment Avoidance (Between-Person) x Novelty and Excitement (Within-Person) | 0.02 | 0.01 | 2.00 | .045 | [0.0004, 0.04] |
| Attachment Avoidance (Between-Person) x Novelty and Excitement (Between-Person) | 0.02 | 0.03 | 0.62 | .536 | [-0.04, 0.08] |
| Attachment Avoidance (Between-Person) x Familiarity and Comfort (Within-Person) | 0.004 | 0.01 | 0.30 | .762 | [-0.02, 0.03] |
| Attachment Avoidance (Between-Person) x Familiarity and Comfort (Between-Person) | 0.13 | 0.04 | 3.84 | <.001 | [0.07, 0.20] |
| Attachment Anxiety (Between-Person) x Novelty and Excitement (Within-Person) | 0.004 | 0.01 | 0.49 | .628 | [-0.01, 0.02] |
| Attachment Anxiety (Between-Person) x Novelty and Excitement (Between-Person) | -0.02 | 0.02 | -0.84 | .403 | [-0.05, 0.02] |
| Attachment Anxiety (Between-Person) x Familiarity and Comfort (Within-Person) | 0.04 | 0.01 | 4.95 | <.001 | [0.03, 0.06] |
| Attachment Anxiety (Between-Person) x Familiarity and Comfort (Between-Person) | 0.04 | 0.03 | 1.43 | .155 | [-0.02, 0.11] |

*Note.* Estimates are derived from a multilevel model in which all predictors in the table were simultaneously entered to predict the outcome. *b* coefficients reflect unstandardized multilevel modeling coefficients.

| **Table S16** | | | | | | | | | | |
| --- | --- | --- | --- | --- | --- | --- | --- | --- | --- | --- |
| ***Study 3 Effects of Attachment Insecurity and Shared Experiences Predicting Daily Reward and Threat (A Path)*** | | | | | | | | | | |
| *Predictors* | *Criterion* | | | | | | | | | |
|  | Relational Reward | | | | | Relational Threat | | | | |
|  | *b* | *SE* | *t* | *p* | 95% CI | *b* | *SE* | *t* | *p* | 95% CI |
| Attachment Avoidance (Between-Person) | -0.10 | 0.03 | -3.67 | <.001 | [-0.15, -0.05] | -0.10 | 0.03 | -3.67 | <.001 | [-0.15, -0.05] |
| Attachment Anxiety (Between-Person) | 0.05 | 0.02 | 2.84 | .005 | [0.02, 0.08] | 0.05 | 0.02 | 2.84 | .005 | [0.02, 0.08] |
| Novelty and Excitement (Within-Person) | 0.06 | 0.01 | 2.84 | <.001 | [0.04, 0.07] | 0.06 | 0.01 | 2.84 | <.001 | [0.04, 0.07] |
| Novelty and Excitement (Between-Person) | 0.02 | 0.02 | 1.21 | .228 | [-0.01, 0.06] | 0.02 | 0.02 | 1.21 | .228 | [-0.01, 0.06] |
| Familiarity and Comfort (Within-Person) | 0.19 | 0.01 | 23.76 | <.001 | [0.17, 0.20] | 0.19 | 0.01 | 23.76 | <.001 | [0.17, 0.20] |
| Familiarity and Comfort (Between-Person) | 0.42 | 0.03 | 14.80 | <.001 | [0.37, 0.48] | 0.42 | 0.03 | 14.80 | <.001 | [0.37, 0.48] |
| Attachment Avoidance (Between-Person) x Novelty and Excitement (Within-Person) | 0.02 | 0.01 | 2.59 | .010 | [0.005, 0.03] | 0.05 | 0.01 | 4.99 | <.011 | [0.03, 0.06] |
| Attachment Avoidance (Between-Person) x Novelty and Excitement (Between-Person) | -0.01 | 0.02 | -0.60 | .547 | [-0.05, 0.03] | -0.01 | 0.02 | -0.60 | .547 | [-0.05, 0.03] |
| Attachment Avoidance (Between-Person) x Familiarity and Comfort (Within-Person) | 0.02 | 0.01 | 2.41 | .016 | [0.004, 0.03] | 0.02 | 0.01 | 2.41 | .016 | [0.004, 0.03] |
| Attachment Avoidance (Between-Person) x Familiarity and Comfort (Between-Person) | 0.06 | 0.02 | 2.40 | .017 | [0.01, 0.11] | 0.06 | 0.02 | 2.40 | .017 | [0.01, 0.11] |
| Attachment Anxiety (Between-Person) x Novelty and Excitement (Within-Person) | -0.01 | 0.01 | -2.07 | .039 | [-0.02, -0.001] | -0.01 | 0.01 | -2.07 | .039 | [-0.02, -0.001] |
| Attachment Anxiety (Between-Person) x Novelty and Excitement (Between-Person) | -0.01 | 0.01 | -0.86 | .390 | [-0.04, 0.01] | -0.01 | 0.01 | -0.86 | .390 | [-0.04, 0.01] |
| Attachment Anxiety (Between-Person) x Familiarity and Comfort (Within-Person) | 0.01 | 0.01 | 1.83 | .067 | [-0.001. 0.02] | 0.01 | 0.01 | 1.83 | .067 | [-0.001. 0.02] |
| Attachment Anxiety (Between-Person) x Familiarity and Comfort (Between-Person) | 0.01 | 0.02 | 0.30 | .768 | [-0.04, 0.05] | 0.01 | 0.02 | 0.30 | .768 | [-0.04, 0.05] |

*Note.* Estimates are derived from a multilevel model in which all predictors in the table were simultaneously entered to predict the outcome. *b* coefficients reflect unstandardized multilevel modeling coefficients.

| **Table S17** | | | | | |
| --- | --- | --- | --- | --- | --- |
| ***Study 3 Effects of Attachment Insecurity and Shared Experiences Predicting Daily Relationship Satisfaction Through Relational Reward and Threat (Direct Effects and B Path)*** | | | | | |
|  | DV: Relationship Satisfaction | | | | |
|  | *Estimate* | *SE* | *t* | *p* | *95% CI* |
| Avoidance (Between-Person) | -0.04 | 0.04 | -1.02 | .309 | [-0.11, 0.04] |
| Anxiety (Between-Person) | -0.03 | 0.03 | -1.35 | .179 | [-0.08, 0.02] |
| Novel and Exciting (Within-Person) | 0.06 | 0.01 | 6.82 | <.001 | [0.05, 0.08] |
| Novel and Exciting (Between-Person) | 0.06 | 0.02 | 2.49 | .014 | [0.01, 0.11] |
| Comfortable and Familiar (Within-Person) | 0.21 | 0.01 | 18.20 | <.001 | [0.18, 0.23] |
| Comfortable and Familiar (Between-Person) | 0.31 | 0.05 | 5.91 | <.001 | [0.21, 0.42] |
| Relational Reward (Within-Person) | 0.42 | 0.02 | 19.73 | <.001 | [0.38, 0.46] |
| Relational Reward (Between-Person) | 0.56 | 0.09 | 6.26 | <.001 | [0.38, 0.73] |
| Relational Threat (Within-Person) | -0.22 | 0.02 | -12.69 | <.001 | [-0.26, 0.19] |
| Relational Threat (Between-Person) | -0.31 | 0.08 | -4.04 | <.001 | [-0.47, -0.16] |
| Avoidance (Between-Person) x Novel and Exciting (Within-Person) | 0.02 | 0.01 | 2.33 | .020 | [0.004, 0.05] |
| Avoidance (Between-Person) x Novel and Exciting (Between-Person) | 0.05 | 0.03 | 1.50 | .134 | [-0.01, 0.11] |
| Avoidance (Between-Person) x Comfortable and Familiar (Within-Person) | 0.002 | 0.01 | 0.19 | .843 | [-0.02, 0.02] |
| Avoidance (Between-Person) x Comfortable and Familiar (Between-Person) | 0.16 | 0.07 | 2.51 | .013 | [0.03, 0.29] |
| Anxiety (Between-Person) x Novel and Exciting (Within-Person) | -0.002 | 0.01 | -0.27 | .785 | [-0.02, 0.01] |
| Anxiety (Between-Person) x Novel and Exciting (Between-Person) | -0.02 | 0.02 | -0.97 | .336 | [-0.05, 0.02] |
| Anxiety (Between-Person) x Comfortable and Familiar (Within-Person) | 0.01 | 0.01 | 1.62 | .105 | [-0.003, 0.03] |
| Anxiety (Between-Person) x Comfortable and Familiar (Between-Person) | 0.001 | 0.04 | 0.03 | .974 | [-0.09, 0.09] |
| Avoidance (Between-Person) x Relational Reward (Within-Person) | -0.01 | 0.02 | -0.44 | .664 | [-0.05, 0.03] |
| Avoidance (Between-Person) x Relational Reward (Between-Person) | -0.15 | 0.09 | -1.68 | .095 | [-0.32, 0.03] |
| Avoidance (Between-Person) x Relational Threat (Within-Person) | 0.01 | 0.02 | 0.42 | .674 | [-0.03, 0.04] |
| Avoidance (Between-Person) x Relational Threat (Between-Person) | 0.02 | 0.07 | 0.24 | .812 | [-0.12, 0.15] |
| Anxiety (Between-Person) x Relational Reward (Within-Person) | 0.12 | 0.02 | 6.58 | <.001 | [0.08, 0.15] |
| Anxiety (Between-Person) Relational Reward (Between-Person) | 0.08 | 0.07 | 1.18 | .238 | [-0.05, 0.21] |
| Anxiety (Between-Person) Relational Threat (Within-Person) | 0.003 | 0.01 | 0.26 | .795 | [-0.02, 0.03] |
| Anxiety (Between-Person) Relational Threat (Between-Person) | 0.06 | 0.05 | 1.14 | .255 | [-0.04, 0.16] |

*Note.* Estimates are derived from a multilevel model in which all predictors in the table were simultaneously entered to predict the outcome. *b* coefficients reflect unstandardized multilevel modeling coefficients.

## **Figure S1.**

## The moderating effect of novelty and excitement on the link between attachment avoidance and daily relationship satisfaction in Studies 1 (A.), 2 (B.), and 3 (C.).

A.

Study 1

Relationship Satisfaction

|  | Low Novel Activities (-1 SD) | High Novel Activities (+1 SD) |
| --- | --- | --- |
| Low Avoidance (-1 SD) | 5.93068 | 6.26818 |
| High Avoidance (+1 SD) | 5.61401 | 6.25613 |

B.

Study 2

Relationship Satisfaction

|  | Low Novel Activities (-1 SD) | High Novel Activities (+1 SD) |
| --- | --- | --- |
| Low Avoidance (-1 SD) | 5.95323 | 6.25509 |
| High Avoidance (+1 SD) | 5.7117 | 6.08249 |

C.

Study 3

Relationship Satisfaction

|  | Low Novel Activities (-1 SD) | High Novel Activities (+1 SD) |
| --- | --- | --- |
| Low Avoidance (-1 SD) | 6.20606 | 6.34589 |
| High Avoidance (+1 SD) | 5.95642 | 6.18443 |

*Note**.* n.s. *p >* .100	**p <* .05	**** p <* .001

**Figure S2.**

The moderating effect of familiarity and comfort on the link between anxiety and daily relationship satisfaction in Studies 1 (A.), 2 (B.) and 3 (C.).

A.

Study 1

Relationship Satisfaction

|  | Low Comfort Activities (-1 SD) | High Comfort Activities (+1 SD) |
| --- | --- | --- |
| Low Anxiety (-1 SD) | 5.63388 | 6.44245 |
| High Anxiety (+1 SD) | 5.4612 | 6.53147 |

B.

Study 2

Relationship Satisfaction

|  | Low Comfort Activities (-1 SD) | High Comfort Activities (+1 SD) |
| --- | --- | --- |
| Low Anxiety (-1 SD) | 5.75241 | 6.43974 |
| High Anxiety (+1 SD) | 5.55345 | 6.25693 |

C.

Study 3

Relationship Satisfaction

|  | Low Comfort Activities (-1 SD) | High Comfort Activities (+1 SD) |
| --- | --- | --- |
| Low Anxiety (-1 SD) | 5.88711 | 6.54589 |
| High Anxiety (+1 SD) | 5.69141 | 6.5684 |

*Note.* n.s. *p* >.100	***p* < .01	*** *p* < .001

**Figure S3.**

The moderating effect of familiarity and comfort on the link between attachment avoidance and daily relationship satisfaction in Studies 1 (A.), 2 (B.), and 3 (C.).

A.

Study 1

Relationship Satisfaction

|  | Low Comfort Activities | High Comfort Activities |
| --- | --- | --- |
| Low Avoidance | 5.58477 | 6.61408 |
| High Avoidance | 5.51031 | 6.35983 |

B.

Relationship Satisfaction

|  | Low Comfort Activities | High Comfort Activities |
| --- | --- | --- |
| Low Avoidance | 5.79908 | 6.40925 |
| High Avoidance | 5.50678 | 6.28742 |

C.

Study 3

Relationship Satisfaction

|  | Low Comfort Activities | High Comfort Activities |
| --- | --- | --- |
| Low Avoidance | 5.89596 | 6.65599 |
| High Avoidance | 5.68255 | 6.4583 |

*Note.* n.s. *p* > .100	† *p* <.100	* *p* < .05	** *p* <.01	*** *p* < .001

**Figure S4.**

The moderating effect of novelty and excitement on the link between attachment anxiety and daily relationship satisfaction in Studies 1 (A.), 2 (B.), and 3 (C.).

A.

Study 1

Relationship Satisfaction

|  | Low Novel Activities | High Novel Activities |
| --- | --- | --- |
| Low Anxiety | 5.82235 | 6.25398 |
| High Anxiety | 5.72234 | 6.27033 |

B.

Study 2

Relationship Satisfaction

|  | Low Novel Activities | High Novel Activities |
| --- | --- | --- |
| Low Anxiety | 5.96749 | 6.22466 |
| High Anxiety | 5.69745 | 6.11292 |

C.

Study 3

Relationship Satisfaction

|  | Low Novel Activities | High Novel Activities |
| --- | --- | --- |
| Low Anxiety | 6.1289 | 6.3041 |
| High Anxiety | 6.03358 | 6.22623 |

*Note.* n.s. p > .100	† p <.100	* p < .05	** p <.01	*** p < .001

**Figure S5.**

The moderating effect of novelty and excitement on the link between attachment avoidance and daily relational reward in Studies 1 (A.) and 3 (B.).

A.

Study 1

Reward

|  | Low Novel Activities | High Novel Activities |
| --- | --- | --- |
| Low Avoidance | 4.51545 | 4.64761 |
| High Avoidance | 4.10418 | 4.46938 |

B.

Study 3

Reward

|  | Low Novel Activities | High Novel Activities |
| --- | --- | --- |
| Low Avoidance | 4.50217 | 4.58906 |
| High Avoidance | 4.28746 | 4.45245 |

*Note.* n.s. *p* > .100	† *p* <.100	* *p* < .05	** *p* <.01	*** *p* < .001

**Figure S6.**

The moderating effect of novelty and excitement on the link between attachment avoidance and daily relational threat in Studies 1 (A.) and 3 (B.).

A.

Study 1

Threat

|  | Low Novel Activities | High Novel Activities |
| --- | --- | --- |
| Low Avoidance | 1.42502 | 1.50464 |
| High Avoidance | 1.50367 | 1.62211 |

B.

*

***

Study 3

Threat

|  | Low Novel Activities | High Novel Activities |
| --- | --- | --- |
| Low Avoidance | 1.35289 | 1.30341 |
| High Avoidance | 1.48848 | 1.62477 |

*Note.* n.s. *p* > .100	† *p* <.100	* *p* < .05	** *p* <.01	*** *p* < .001

**Figure S7.**

The moderating effect of familiarity and comfort on the link between attachment avoidance and daily relational reward in Studies 1 (A.) and 3 (B.).

A.

Study 1

Reward

|  | Low Comfort Activities | High Comfort Activities |
| --- | --- | --- |
| Low Avoidance | 4.36546 | 4.7976 |
| High Avoidance | 4.08355 | 4.49001 |

B.

Study 3

Reward

|  | Low Comfort Activities | High Comfort Activities |
| --- | --- | --- |
| Low Avoidance | 4.37778 | 4.71345 |
| High Avoidance | 4.16787 | 4.57204 |

*Note.* n.s. *p* > .100	† *p* <.100	* *p* < .05	** *p* <.01	*** *p* < .001

**Figure S8.**

The moderating effect of familiarity and comfort on the link between attachment avoidance and daily relational threat in Studies 1 (A.) and 3 (B.).

A.

Study 1

Threat

|  | Low Comfort Activities | High Comfort Activities |
| --- | --- | --- |
| Low Avoidance | 1.59439 | 1.33527 |
| High Avoidance | 1.63203 | 1.49375 |

B.

Study 3

Threat

|  | Low Comfort Activities | High Comfort Activities |
| --- | --- | --- |
| Low Avoidance | 1.47682 | 1.17948 |
| High Avoidance | 1.64769 | 1.46556 |

*Note.* n.s. *p* > .100	† *p* <.100	* *p* < .05	** *p* <.01	*** *p* < .001

**Figure S9.**

The moderating effect of familiarity and comfort on the link between attachment anxiety and daily relational threat in Studies 1 (A.) and 3 (B.).

A.

Study 1

Threat

|  | Low Comfort Activities | High Comfort Activities |
| --- | --- | --- |
| Low Anxiety | 1.51677 | 1.3027 |
| High Anxiety | 1.70965 | 1.52632 |

B.

Study 3

Threat

|  | Low Comfort Activities | High Comfort Activities |
| --- | --- | --- |
| Low Anxiety | 1.42452 | 1.16699 |
| High Anxiety | 1.69999 | 1.47806 |

*Note.* n.s. *p* > .100	† *p* <.100	* *p* < .05	** *p* <.01	*** *p* < .001

**Figure S10.**

The moderating effect of familiarity and comfort on the link between attachment anxiety and daily relational reward in Studies 1 (A.) and 3 (B.).

A.

Study 1

Reward

|  | Low Comfort Activities | High Comfort Activities |
| --- | --- | --- |
| Low Anxiety | 4.25765 | 4.67025 |
| High Anxiety | 4.19136 | 4.61736 |

B.

Study 3

Reward

|  | Low Comfort Activities | High Comfort Activities |
| --- | --- | --- |
| Low Anxiety | 4.22449 | 4.56682 |
| High Anxiety | 4.32116 | 4.71867 |

*Note.* n.s. *p* > .100	† *p* <.100	* *p* < .05	** *p* <.01	*** *p* < .001

**Figure S11.**

The moderating effect of novelty and excitement on the link between attachment anxiety and daily relational reward in Studies 1 (A.) and 3 (B.).

A.

Study 1

Reward

|  | Low Novel Activities | High Novel Activities |
| --- | --- | --- |
| Low Anxiety | 4.36955 | 4.55835 |
| High Anxiety | 4.25008 | 4.55863 |

B.

Study 3

Reward

|  | Low Novel Activities | High Novel Activities |
| --- | --- | --- |
| Low Anxiety | 4.31689 | 4.47442 |
| High Anxiety | 4.47274 | 4.56709 |

*Note.* n.s. *p* > .100	† *p* <.100	* *p* < .05	** *p* <.01	*** *p* < .001

**Figure S12.**

The moderating effect of novelty and excitement on the link between attachment anxiety and daily relational threat in Studies 1 (A.) and 3 (B.).

A.

Study 1

Threat

|  | Low Novel Activities | High Novel Activities |
| --- | --- | --- |
| Low Anxiety | 1.35785 | 1.39824 |
| High Anxiety | 1.53517 | 1.69024 |

B.

Study 3

Threat

|  | Low Novel Activities | High Novel Activities |
| --- | --- | --- |
| Low Anxiety | 1.24331 | 1.34819 |
| High Anxiety | 1.59806 | 1.57999 |

*Note.* n.s. *p* > .100	† *p* <.100	* *p* < .05	** *p* <.01	*** *p* < .001

# **Validation Study**

We conducted a separate validation study with two key goals: 1) to test for the distinctiveness of our key constructs and 2) to obtain and code examples of familiar and comfortable and novel and exciting people report engaging in with their partner. In a sample recruited on CloudResearch, we collected data from a total of 315 participants. After excluding those who did not meet the inclusion criteria (*n* = 34) and failed the attention check question (*n* = 3), 278 participants were included in the analysis. The average age of participants was 47.36 years (*SD* = 10.74), while the average relationship length was 157.97 months (*SD* = 107.59). In total, 134 men, 146 women, and 1 non-binary participant were included in the study. the majority of participants identified as straight/heterosexual (*n* = 238), white (*n* = 206), married (*n* = 210) and as having completed a 4-year university degree (*n* = 117).

# **Testing the Distinctiveness of our Key Constructs**

Given the high correlations between several of our key constructs (e.g., familiar and comfortable experiences, novel and exciting experiences, relational reward, and relationship satisfaction), we conducted a series of confirmatory factor analysis (CFA) models to test whether constructs load together on one factor (i.e., assess the same construct) or load on two separate factors (i.e., assess different constructs). To do this, we examined the model fit indices for each model (i.e., SRMR < .08; CFI > 0.90; TLI > .90; RMSEA < .08; lower BIC value) and conducted chi-square difference tests to determine if one model fit significantly better. All analyses were performed in R using the *dplyr* (Wickham et al., 2023), *lavaan* (Rosseel, 2012), and *semPlot* (Epskamp, 2022) packages. Below, see the variable item list and the fit indices for each comparison.

**Variable-Item List**

| **Comfortable-familiar Experiences** | **Novel-exciting Experiences** | **Relationship Satisfaction** | **Reward** |
| --- | --- | --- | --- |
| How much did your partner provide you with a source of comfort and stability? (com1) | How much did being with your partner result in you having new experiences? (novel1) | How satisfied were you with your relationship? (sat1) | I loved my partner a lot. (rew1) |
| Did you engage in activities that felt familiar and comfortable with your partner? (com2) | Did you feel a greater awareness of things because of your partner? (novel2) | How content were you with your relationship? (sat2) | I had strong, positive feelings for my partner. (rew2) |
| I enjoyed doing ordinary things with my partner. (com4) | How much did being with your partner expand your sense of the kind of person you are? (novel3) | How happy were you with your relationship? (sat3) | I felt closer with my partner than I've ever felt to somebody. (rew) |
|  | How much did your partner provide you with a source of excitement? (novel4) |  |  |
|  | How much did you feel you gained a larger perspective on things because of your partner? (novel5) |  |  |
|  | How much did your partner increase your knowledge? (novel6) |  |  |

**Testing the Distinction Between Familiar-Comfortable Experiences and Relationship Satisfaction**

|  | 2-Factor | 1-Factor |
| --- | --- | --- |
| Comparative Fit Index (CFI) | 0.980 | 0.935 |
| Tucker-Lewis Index (TLI) | 0.962 | 0.892 |
| Akaike (AIC) | 3740.203 | 3832.447 |
| Bayesian (BIC) | 3787.362 | 3875.978 |
| Sample-size adjusted Bayesian (SABIC) | 3746.140 | 3837.927 |
| RMSEA | 0.137 | 0.232 |
| SRMR | 0.034 | 0.075 |
| Chi-Square (Model test user model test statistic) | 49.841 | 144.085 |

The comparison between the 2-factor and 1-factor models (Chi-squared difference test) showed that the 2-factor model provided a significantly better fit than the 1-factor model (*p* < .001).

**Testing the Distinction Between Novel-Exciting Experiences and Relationship Satisfaction**

|  | 2-Factor | 1-Factor |
| --- | --- | --- |
| Comparative Fit Index (CFI) | 0.960 | 0.583 |
| Tucker-Lewis Index (TLI) | 0.945 | 0.445 |
| Akaike (AIC) | 6749.298 | 7828.443 |
| Bayesian (BIC) | 6818.016 | 7893.545 |
| Sample-size adjusted Bayesian (SABIC) | 6757.771 | 7836.471 |
| RMSEA | 0.126 | 0.401 |
| SRMR | 0.052 | 0.144 |
| Chi-Square (Model test user model test statistic) | 140.378 | 1221.524 |

The comparison between the 2-factor and 1-factor models (Chi-squared difference test) showed that the 2-factor model provided a significantly better fit than the 1-factor model (*p* < .001).

**Testing the Distinction Between Reward and Relationship Satisfaction**

|  | 2-Factor | 1-Factor |
| --- | --- | --- |
| Comparative Fit Index (CFI) | 0.993 | 0.943 |
| Tucker-Lewis Index (TLI) | 0.987 | 0.905 |
| Akaike (AIC) | 3458.113 | 3582.617 |
| Bayesian (BIC) | 3505.225 | 3626.105 |
| Sample-size adjusted Bayesian (SABIC) | 3464.004 | 3588.055 |
| RMSEA | 0.088 | 0.239 |
| SRMR | 0.019 | 0.038 |
| Chi-Square (Model test user model test statistic) | 25.196 | 151.700 |

The comparison between the 2-factor and 1-factor models (Chi-squared difference test) showed that the 2-factor model provided a significantly better fit than the 1-factor model (*p* < .001).

**Testing the Distinction Between Familiar-Comfortable Experiences and Reward**

|  | 2-Factor | 1-Factor |
| --- | --- | --- |
| Comparative Fit Index (CFI) | 0.959 | 0.933 |
| Tucker-Lewis Index (TLI) | 0.923 | 0.889 |
| Akaike (AIC) | 4220.063 | 4257.134 |
| Bayesian (BIC) | 4267.175 | 4300.622 |
| Sample-size adjusted Bayesian (SABIC) | 4225.954 | 4262.571 |
| RMSEA | 0.168 | 0.201 |
| SRMR | 0.035 | 0.046 |
| Chi-Square (Model test user model test statistic) | 70.274 | 109.344 |

The comparison between the 2-factor and 1-factor models (Chi-squared difference test) showed that the 2-factor model provided a significantly better fit than the 1-factor model (*p* < .001).

**Testing the Distinction Between Novel-Exciting Experiences and Reward**

|  | 2-Factor | 1-Factor |
| --- | --- | --- |
| Comparative Fit Index (CFI) | 0.954 | 0.717 |
| Tucker-Lewis Index (TLI) | 0.936 | 0.623 |
| Akaike (AIC) | 7275.310 | 7795.431 |
| Bayesian (BIC) | 7343.959 | 7860.467 |
| Sample-size adjusted Bayesian (SABIC) | 7283.715 | 7803.393 |
| RMSEA | 0.120 | 0.290 |
| SRMR | 0.062 | 0.136 |
| Chi-Square (Model test user model test statistic) | 128.119 | 650.240 |

The comparison between the 2-factor and 1-factor models (Chi-squared difference test) showed that the 2-factor model provided a significantly better fit than the 1-factor model (*p* < .001).

In sum, across all comparisons, we find evidence that the constructs are distinct (i.e., load onto separate factors). We should also note that although the RMSEA was greater than .08 in all models, the results indicated that this value was not significantly greater than .08 for any of the models (p > .05)

# **Coding Examples of Familiar-Comfortable and Novel-Exciting Experiences**

Our second goal in collecting additional data was to collect examples of novel-exciting and familiar-comfortable activities that people engage in with their partner. Specifically, for novel-exciting activities, after participants completed the measure of self-expansion, we asked: “*Thinking of your responses to the questions above, can you think of an example of a novel and exciting activity you engaged in with your partner? Please describe it in the space below*.” We asked the same question for familiar-comfortable activities. At the end of the survey, we also asked whether participants had used any artificial intelligence tools (e.g., ChatGPT), noting that doing so would not affect their compensation. After excluding participants who reported using artificial intelligence (*n* = 3), those who did not meet the study’s inclusion criteria (i.e., being in a romantic relationship of at least two years and living with their partner; *n* = 34), and failed the attention check question (*n* = 3), our final sample for the coding the open-ended responses included 275 participants.

**Coding of the Open-Ended Responses**

To code the responses, we used thematic analyses (Braun & Clarke, 2023) and were guided by an adapted version of themes identified by Muise and colleagues (2019) for coding novel and exciting experience, which we also extended to familiar and comfortable experiences. Coding was conducted by the third author, and any uncertainties were resolved in consultation with the second and last authors. In addition, the second and last authors reviewed the coding for accuracy.

We report the frequency of the codes in Table S17 below. For novel–exciting activities, the majority of participants provided examples of special events they engaged in outside the home, such as vacations or dinners at special locations. The second most common category included activities in which they learned something new with or from their partner, such as taking a dance class or learning to change a tire. Participants also mentioned engaging in physical activities, such as hiking, rafting, or ziplining. For familiar-comfortable activities, the most common examples involved leisure activities at home with their partner, such as watching favorite television shows together while cuddling or having dinner at home. These were followed by household activities, such as cooking together, cleaning, or going grocery shopping. The third most common category included outings, such as going to the cinema or visiting the park.

**Coding for Similarity Between Experiences**

We also coded whether participants provided similar examples for both novel-exciting and comfortable-familiar activities. To do this, we compared each participant’s responses for both activity types and coded whether they were similar. Only 9 out of the 275 participants provided similar examples for both categories. For instance, one participant described painting a canvas as both a novel-exciting and a comfortable-familiar activity but also listed visiting the beach and trying new foods as novel-exciting activities. Another participant described playing games as both novel-exciting and comfortable-familiar. These were coded as similar, but the participant specified that the novel-exciting example involved playing “weird”, “niche”, and “co-op” games together, whereas the comfortable-familiar example involved playing UNO. In summary, participants’ examples for novel–exciting and familiar-comfortable experiences generally differed. Even when the activities were similar, sub-characteristics of the activity (e.g., type of game) influenced whether it was perceived as novel-exciting or comfortable-familiar.

***Themes, Definitions and Frequency of Themes for Novel-Exciting and Familiar-Comfortable Experiences***

| **Themes** | **Definition of Theme** | **Total Score for Novel-Exciting** | **Total Score for Familiar-Comfortable** |
| --- | --- | --- | --- |
| Outings | Attending a special event outside or having a date night with a partner | 151 | 39 |
| Household activities | Completing household or maintenance tasks with a partner | 13 | 59 |
| Leisure | Spending time with a partner that did not include a special event or outing; relaxing together or passive activities such as watching television | 24 | 166 |
| Disclosure | Disclosing thoughts and feelings to a partner; having an in-depth conversation | 13 | 22 |
| Future | Planning or discussing future events relevant to the relationship; planning a wedding, shopping for a new house, discussing future life events (i.e., children, honeymoon) | 7 | 0 |
| Sex | Engaging in sexual activity with one’s partner | 1 | 0 |
| Care | Giving or receiving care or support including practical, emotional support, or parenting. | 14 | 10 |
| Physical activity | Doing something active with a partner such as exercising, playing a sport, or participating in outdoor physical activities | 46 | 25 |
| Affection | Cuddling or being intimate with a partner that is not directly associated with sex | 4 | 20 |
| Learning | Learning something new with a partner, teaching a partner something, or being taught something by a partner | 95 | 0 |
| Negative | Going through negative event together. | 3 | 0 |
| Religious/spiritual/ethnic | Attending a religious service or engaging in spiritual/ethnic activities with a partner such as praying or meditating | 3 | 4 |
| Humor | Laughing or sharing a funny experience with a partner | 32 | 13 |
| Life transition | A major life transition that significantly changes their life (e.g., relocation, having a child, etc.) | 11 | 0 |

Similar = 9

# **References**

Braun, V., & Clarke, V. (2023). Thematic analysis. In H. Cooper, M. N. Coutanche, L. M. McMullen, A. T. Panter, D. Rindskopf, & K. J. Sher (Eds.), APA handbook of research methods in psychology: Research designs: Quantitative, qualitative, neuropsychological, and biological (2nd ed., pp. 65–81). American Psychological Association. [https://doi.org/10.1037/0000319-004](https://psycnet.apa.org/doi/10.1037/0000319-004)

Epskamp, S. (2022*). semPlot: Path diagrams and visual analysis of various SEM packages’ output* (R package version 1.1.6). Comprehensive R Archive Network (CRAN). https://CRAN.R-project.org/package=semPlot

Muise, A., Harasymchuk, C., Day, L. C., Bacev-Giles, C., Gere, J., & Impett, E. A. (2019). Broadening your horizons: Self-expanding activities promote desire and satisfaction in established romantic relationships. *Journal of Personality and Social Psychology*, *116*(2), 237. http://dx.doi.org/10.1037/pspi0000148

Rosseel, Y. (2012). lavaan: An R package for structural equation modeling. *Journal of Statistical Software, 48*(2), 1–36. https://doi.org/10.18637/jss.v048.i02

Wickham, H., François, R., Henry, L., & Müller, K. (2023). *dplyr: A grammar of data manipulation* (R package version 1.1.4). Comprehensive R Archive Network (CRAN). <https://CRAN.R-project.org/package=dply>
